# Mucosal immune cell priming by intranasally delivered *Haemophilus haemolyticus* is associated with heterologous protection against influenza and nontypeable *Haemophilus influenzae*

**DOI:** 10.3389/fimmu.2026.1855464

**Published:** 2026-07-20

**Authors:** Jack S. Pepper, Caitlyn M. Granland, Sharon L. Clark, Ruth B. Thornton, Josephine Bayliss, M. Z. Edison Foo, Wesley Billingham, Naomi Scott, Alma Fulurija, Deborah H. Strickland, Selma P. Wiertsema, Peter C. Richmond, Elke J. Seppanen, Lea-Ann S. Kirkham, M. Christian Tjiam

**Affiliations:** 1 Wesfarmers Centre of Vaccines and Infectious Diseases, The Kids Research Institute Australia, Perth, WA, Australia; 2 Centre for Child Health Research, The University of Western Australia, Perth, WA, Australia; 3 Biostatistics, The Kids Research Institute Australia, Perth, WA, Australia; 4 School of Biomedical Sciences, The University of Western Australia, Perth, WA, Australia; 5 Medical, Molecular and Forensic Sciences, Murdoch University, Perth, WA, Australia; 6 Wal-yan Respiratory Research Centre, The Kids Research Institute Australia, Perth, WA, Australia; 7 BioscienceConnect, Utrecht, Netherlands; 8 Discipline of Paediatrics, Medical School, The University of Western Australia, Perth, WA, Australia; 9 Immunology and General Paediatrics Departments, Perth Children’s Hospital, Perth, WA, Australia

**Keywords:** immune priming, influenza A, intranasal vaccination, lungs, nasal tissue, otitis media

## Abstract

**Introduction:**

Intranasal vaccines offer a needle-free strategy to enhance immunity to respiratory infections. We investigated the mechanism of action of a novel intranasal vaccine using the human respiratory commensal *Haemophilus haemolyticus* (Hh), previously shown to protect against nontypeable *Haemophilus influenzae* (NTHi) otitis media and accelerate clearance of influenza A virus (IAV).

**Methods:**

Mucosal and systemic cellular immune responses were assessed 2–144 hours after intranasal Hh treatment in mice, compared with placebo or the Toll-Like Receptor (TLR)2–6 agonist Pam2CSK4 using spectral flow cytometry. The impact of treatment on subsequent IAV and NTHi challenge was also evaluated.

**Results:**

Hh induced a distinct, tissue-specific immune signature with rapid recruitment of neutrophils and inflammatory monocytes to the lungs, peaking at 6 hours, earlier than Pam2CSK4. Hh also generated higher proportions of nasal CD103^+^CD4^+^ T cells within 48 hours, which further expanded following sequential IAV and NTHi infection.

**Discussion:**

These findings demonstrate that Hh primes mucosal immune responses to promote heterologous protection.

## Introduction

Approximately 12 billion upper respiratory tract infections (URTIs) occur globally each year, these are mostly viral and with the highest incidence rates in young children and older adults (1). Bacterial middle ear infections (otitis media, OM) and lower respiratory tract infections such as pneumonia are common complications of URTIs, the latter among the leading infectious causes of death worldwide (1). URTIs, OM and pneumonia drive substantial antibiotic use, particularly in primary care, where overuse and/or prolonged courses accelerate antimicrobial resistance (AMR) (2). AMR was estimated to cause 4.71 million deaths in 2021 and predicted to cause 8.22 million deaths by 2050 (3). Next−generation inhaled and intranasal biologics, including vaccines, offer a promising strategy to elicit heterotypic, mucosal immunity against viral and bacterial respiratory pathogens (4, 5) that would improve infection prevention and reduce antibiotic reliance.

Host permissibility to bacterial respiratory infections including bacterial OM and pneumonia is enhanced with prior or concurrent respiratory virus infection (6, 7). A major causative pathogen of OM and community acquired pneumonia is nontypeable *Haemophilus influenzae* (NTHi) (8–10), which colonises the nasopharynx and, when triggered by viral infection, can ascend the Eustachian tube to infect the middle ear or disseminate into the lungs. To mimic the clinical pathogenesis of OM, murine and chinchilla models of OM have used viral infections to drive the ascension of multiple species of bacteria into the ears or to descend into the lungs (11–13).

Administration of live bacteria, or bacterial pathogen associated molecular patterns (PAMPs), is efficacious against homologous and heterologous infectious challenge. The Toll-like receptor (TLR) 2/6 agonist INNA-051 administered intranasally accelerates clearance of influenza A virus (IAV) in a human challenge model (14). INNA-051 reduced bronchoalveolar neutrophil frequencies and Keratinocyte-derived Cytokine (KC) following Rhinovirus A1 challenge in mice (15). Similarly, the intranasal BPZE1 vaccine comprising of genetically detoxified *Bordetella pertussis* reduced pro-inflammatory cytokines and mortality due to IAV challenge in mice (4, 16). Intravenous *Mycobacterium bovis* demonstrated subsequent protection of mice from SARS-CoV-2 infection in an IFNγ-dependent manner (17). These findings demonstrate a pivotal role of bacterial immune priming in enhancing host resilience to heterologous challenge.

Building on this emerging field, we have shown that intranasal administration of *Haemophilus haemolyticus* (Hh), a human respiratory commensal that is genotypically related to NTHi, prevents development of NTHi-OM in the IAV-NTHi OM mouse model and accelerates clearance of IAV from the lungs (13). From these findings, we are developing an intranasal vaccine containing live Hh to prevent OM in children. In mice, Hh treatment reduced inflammatory cytokine responses (Interleukin(IL)-6 and KC) to IAV infection (13), which may be critical for its capability to prevent influenza-driven NTHi-OM. While most bacterial priming strategies involve challenge with unrelated bacterial or viral organisms, Hh and NTHi share 92-94% sequence homology that may promote cross-reactive immunity (18), where mucosal antibody and memory lymphocyte responses may contribute to protection.

The cellular immune dynamics following intranasal administration of commensal bacteria – including Hh – remains uncharacterised and is critical to advance novel vaccines that afford heterologous mucosal protection. Here, we aimed to define respiratory-specific and systemic immunity in mice treated with intranasal Hh and challenged with IAV and NTHi, using Pam2CSK4 as a comparator to investigate how a whole organism formulation (Hh) differs in the immune response to a purified TLR2/6 ligand, previously shown to protect mice from respiratory pathogen challenge (19). We hypothesised that Hh treatment will induce an early innate cellular response distinct from that of purified TLR ligands, and that Hh will modulate cellular immunity upon subsequent IAV and NTHi challenge. For the first time, we describe cellular features induced by Hh treatment that are associated with protection from influenza and NTHi.

## Materials and methods

### Microbial strains

*H. haemolyticus* strain H19 was isolated, species confirmed, and grown, as described (13, 20, 21). Prior to use, vials were thawed, washed 3 times (16,000g for 3 minutes) and resuspended in phosphate buffered saline (PBS) pH7.4 (Gibco, USA) to a final concentration of 5x10^9^ colony forming units [CFU]/mL.

Influenza A (Memphis/1/71) H3N2 virus inoculum was prepared as described (18). Viral titres were determined by plaque assay to derive plaque-forming units [PFU]/mL.

Nontypeable *Haemophilus influenzae* strain R2866 Spec^r^ was prepared as described (18) using 0.1mg/mL spectinomycin to maintain resistance. All bacterial and viral stocks were stored at -80 °C as single-use inoculum.

### Ethics and mice

This study was approved by The Kids Research Institute Australia Animal Ethics Committee (Perth, Australia; AEC#2299). ARRIVE 2.0 guidelines were followed for best practice in experimental design, implementation, and reporting (**Supplementary Table 4**). Pathogen-free BALB/cJ mice (in-house colony at The Kids Research Institute Australia, Perth, Australia) aged 6–8 weeks were housed under Physical Containment level 2 in a 12:12 standard light/dark cycle and acclimatised for ≥3 days. Experiments were conducted in sets of six mice, per treatment, per timepoint (exact numbers per study arm are detailed in **Supplementary Table 1**). Sample sizes were calculated based on preliminary nasal wash KC data. Male and female (50.69%:49.31%) mice were randomised to treatment group using a random number generator. Environmental enrichment included nesting material, tunnels, and gnawing blocks. Laboratory and analysis personnel were not blinded to group assignments. Mice were excluded if they sustained any injury due to agonistic behaviour (biting, *etc.*). No mice or data were excluded from the analysis.

### Treatments

**Figure 1:**
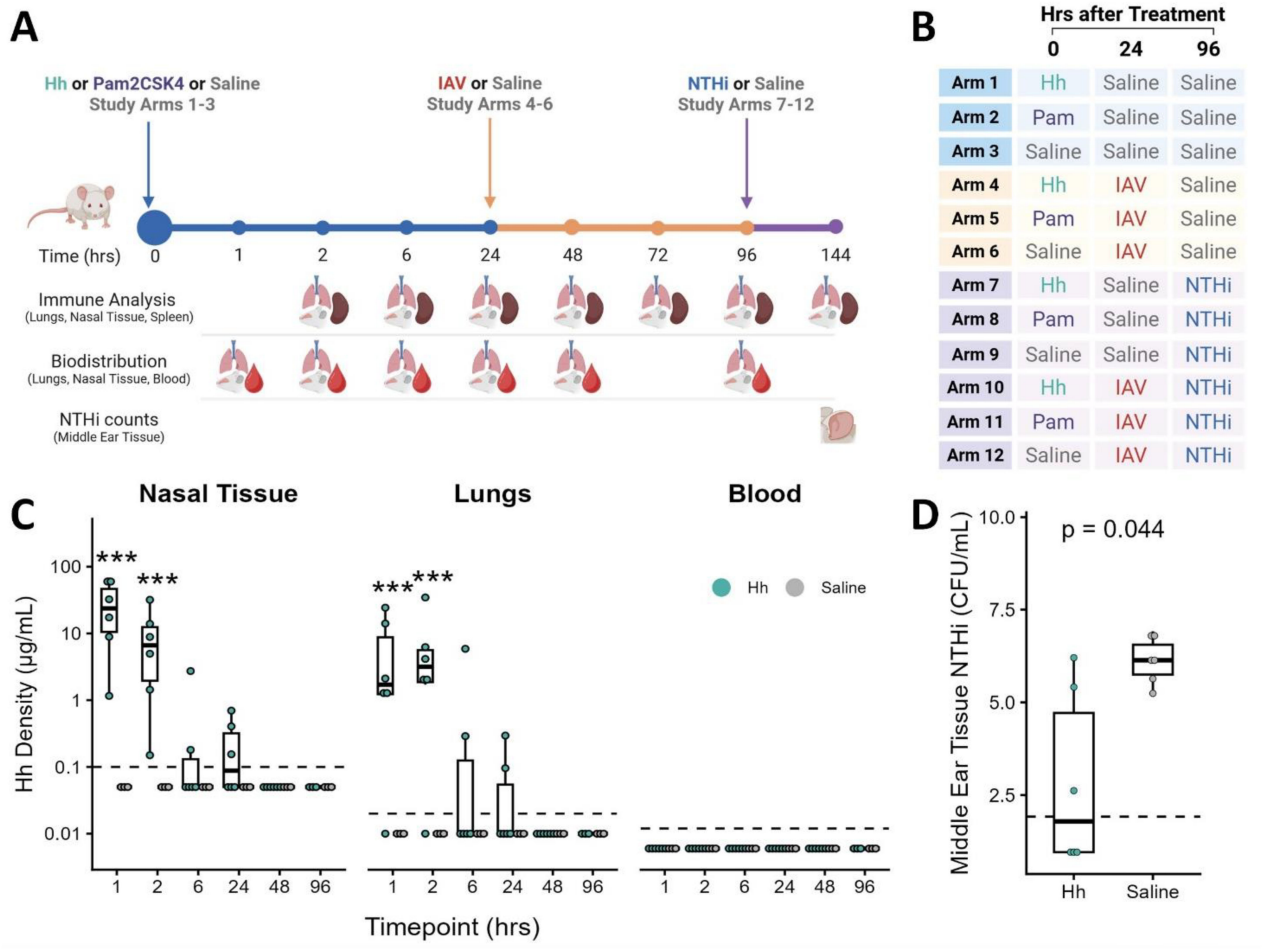
Experimental design, Hh biodistribution, and NTHi density in ear. **(A)** Experimental timeline, including schedules of treatments, infectious challenges and tissue types harvested for immunology, biodistribution and NTHi middle ear tissue quantification experiments. **(B)** Study arms comprised of unique combinations of treatments, controls, and infectious challenges. **(C)** Hh quantitative PCR analysis of tissues demonstrated that intranasally delivered Hh is restricted to respiratory tissue distribution and cleared within 24–48 hours. Dashed line indicates the limit of detection (Nasal tissue, 0.1pg/mL; Lungs, 0.02pg/mL; Blood, 0.012pg/mL). n=6 per timepoint for Hh, n=3 per timepoint for Saline. **(D)** Hh treatment reduces density of NTHi in middle ear tissue collected at 144 hours in the IAV+NTHi challenge model. Dashed line indicates the limit of detection (1.92 Log_10_CFU/mL). Two-way ANOVA-style linear model with estimated marginal means contrast was used to compare Hh distribution across various timepoints and tissues. Wilcoxon signed-rank test used to compare Hh and Saline on NTHi colonisation of Middle Ear Tissue. CFU, colony-forming units; Hh, Haemophilus haemolyticus; hrs, hours; IAV, influenza A virus; NTHi, nontypeable Haemophilus influenzae; Pam, Pam2CSK4. Created in BioRender. Tjiam, C. (2026) https://BioRender.com/5mxh5mq. *** represents p value < 0.001.

Mice were lightly anaesthetised (1L/min 5% v/v isoflurane in room air using the Somnosuite Anaesthetic system; APAC Scientific, AU) and administered either 10μL intranasal Hh (5x10^7^ CFU in saline), Pam2CSK4 (1μg/mL in saline; InVivogen, USA), or saline-only placebo (**Figure 1A**). The Pam2CSK4 dosage was chosen based on preliminary *in vivo* titration that demonstrated 1µg dose to be most similar to Hh in safety (weight, clinical scores) and induction of lung neutrophils (data not shown). In the IAV-NTHi infection model, anaesthetised mice were administered 25µL intranasal IAV (1x10^4.5^ PFU in saline) at 24h post-Hh, and 10µL intranasal NTHi (5x10^7^ CFU in saline) 96h post-Hh. Mice were inoculated in ascending cage order to minimise confounders and monitored daily.

### Tissue collection and processing

At collection times of 2, 6, 24, 48, 72, 96 and 144 hours post-treatment, mice were euthanised with intraperitoneal ketamine (400mg/kg) and xylazine (40mg/kg) and perfused with 10mL saline containing 100U/mL heparin via the left ventricle. Lungs, spleen, and nasal tissue were collected post-mortem in RPMI, on ice, for up to 2h. Lungs and nasal tissue were disaggregated using a sterile scalpel, then enzymatically dissociated with collagenase type 4 (Worthington, USA; 1μg/mL for nasal tissue and 2μg/mL for lungs) and DNase 1 (Merck, USA; 7.5μg/mL for nasal tissue and 15μg/mL for lungs) under continuous agitation at 37 °C for 60 minutes. For lungs, additional DNase 1 (2µg/mL) was added for a further 30 minutes at 37 °C, filtered through a 70µm nylon filter (Corning, USA) and washed with 1x PBS (Gibco, USA). Spleens were injected with 1.5mL 1% w/v BSA/PBS (Bovine Serum Albumin: Merck, USA) using a 22-gauge syringe, mechanically dissociated with a scalpel, filtered through a metal mesh, then a 70µm nylon filter, and washed with 30mL 1% w/v BSA/PBS. Plasticware and filters were pre-rinsed with filter-sterilised heat-inactivated fetal bovine serum (Gibco) to minimise cell retention. Samples were resuspended in 2mL ACK lysis buffer (Gibco, USA) for 3 minutes at room temperature with constant agitation, washed in 20mL PBS, and enumerated in a haemocytometer.

Batch Quality Control samples were collected prior to study start. These comprised solely of spleens collected from untreated mice. Spleens were processed as above, and cryopreserved in FBS supplemented with 10% DMSO and stored in liquid nitrogen, until required.

### Full-spectrum flow cytometry

A full spectrum flow cytometry panel was developed to probe major innate and adaptive immune subsets in BALB/c mice (antibody clones, fluorochrome conjugates and concentrations are detailed in **Supplementary Table 2**). Panel development involved characterisation of target tissue autofluorescence profiles, strategic assignment of previously validated antibody clones (22–24) to fluorochromes (based on marker density and co-expression profiles), antibody titration and peak-detector photomultiplier tube gain optimisations to identify reagent concentrations and instrument settings that maximised marker resolution. Spectral flow cytometry was performed on a 5-laser, 48-detector FACSymphony™ A5 SE (BD Biosciences) operating FACSDiva™ Software (v9.3.1). PMT voltages were calibrated specifically for this panel; optimised peak-detector voltages were appended to default (in-house calibrated with mid-fluorescence beads) detector settings. Unmixing was performed in FACSDiva™ using single-stained UltraComp eBeads and unstained cells (lungs, spleen, nasal tissue) to capture tissue autofluorescence profiles. Unmixing accuracy was empirically verified by evaluating single-stained cells deconvoluted with the first pass mixing matrix, to identify fluorochrome unmixing error(s) presenting as false positives or extreme negatives in other parameters (encompassing *N* × *N* comparisons). Fluorochrome unmixing errors were amended by reacquiring single-stained cells in place of beads for problematic dyes, to provide cell-accurate spectra where bead-derived spectra were inaccurate. A new mixing matrix was generated with the new controls and empirically verified a final time to confirm accuracy.

1x10^6^ cells from each tissue were sampled, washed with 2mL PBS, then incubated with 1mL FVS440UV (diluted 1:2000; BD Biosciences, San Jose, CA) for 15 minutes in the dark at room temperature. Cells were washed with 1% BSA/PBS. An antibody cocktail at pre-titred concentrations (**Supplementary Table 2**) was prepared fresh, centrifuged at 10,000g for 4 minutes, and clarified cocktail (supernatant) was added to each sample, including to a batch control (comprising a cryopreserved pool of BALB/c splenocytes, run in every experiment), for 15 minutes in the dark at room temperature, washed twice with 1% BSA/PBS, fixed with cold 1% Paraformaldehyde/PBS (Merck, USA) for 5 minutes at room temperature, washed with 1% BSA/PBS, and acquired within 24 hours. Prior to acquisition, instrument quality control was performed within FACSDiva™ using Cytometer Setup & Tracking beads (BD Biosciences) to ensure cytometer performance passed acceptance criteria (comparing ΔPMT voltage and %CV, for all detectors, relative to baseline). Raw data was exported as FCS3.0 files for analysis.

### Computational flow cytometry analysis packages

Computational analysis of data was conducted using the packages *CytoNorm* (v2.0.1), *Spectre* (v1.2.0)^1^, *RPhenoannoy* (v0.1.0)^2^, *UWOT* (v0.2.2)^3^, and *RcppHNSW* (v0.6.0)^4^ using R (v4.4.0) (Rstudio – v2024.04.0+735).

### Pre-processing of flow data

FCS3.0 data files were imported into FlowJo v10.10.0 (Becton Dickinson). Data was cleaned for downstream computational analyses by excluding signal deviations (parameters vs time), doublets, cells with high autofluorescence, FVS440UV^+^ or CD45^-^ cells (gating strategy shown in **Supplementary Figure 6**).

### Batch alignment of flow data

*CytoNorm* was used to train an alignment model using Batch Quality Controls included within each experiment (50,000 events/QC; total 2.05x10^6^ events) using default parameters except number of clusters (nClus = 22). The trained normalisation model was applied to pre-processed flow data, to batch-align signals between experiments.

### High dimensional flow cytometry analysis

Batch-aligned data were Logicle-transformed with *Spectre*. Clustering was performed using *RPhenoannoy* using default parameters except number of nearest neighbours (k = 40). Uniform Manifold Approximation and Projection (UMAP) was performed using the *UWOT* package, with default parameters except nearest neighbours (n_neighbors = 30; nn_method = “hnsw”). All parameters except Live/Dead, CD45^+^, and Auto-fluorescence 1 and 2 were included the analyses. Summaries of cell cluster annotations are summarised in **Supplementary Table 3**. Cell frequencies were calculated as a percentage of all live, CD45^+^ events per sample.

### Tissue processing for quantitative PCR

Nasal tissue, lungs and blood were collected at 1h, 2h, 6h, 24h, 48h, 96h post-treatment. Blood was stored on ice, before centrifugation at 16,000g for 3 minutes to remove serum, and the blood pellet was snap frozen. Nasal tissue and lungs were snap frozen then homogenised using a plastic pestle. In a fresh tube, 100mg of homogenised tissue was lysed overnight in proteinase K (1μL/mg of tissue) and ATL buffer for a final concentration of 100mg tissue/mL. The following day, 200mg of lysed tissue was transferred to a new tube to continue with extractions the QIAamp DNA Mini Kit (Qiagen). Blood pellets were thawed and reconstituted to the original blood collection volume with 1x PBS (Gibco). 200μL of resuspended blood was lysed with 20μL proteinase K for 3 hours, with extractions continuing with the Qiagen QIAamp DNA Mini Kit. DNA extractions were performed according to manufacturer’s instructions (13).

### *Haemophilus haemolyticus* qPCR

Quantitative polymerase chain reaction (qPCR) was performed on the CFX96 system (Bio-Rad, USA) as described in Scott et al. (13) to detect the *Haemophilus haemolyticus*-specific *hyp*D gene (25) using primers *hyp*D-F (5′-GGCAATCAGATGGTTTACAACG), *hyp*D-R (5′-CAGCTTAAAGYAAGYAGTGAATG) (Sigma), and *hyp*D-Probe (5′-VIC-CCACAACGAGAATTAG-MGBNFQ; Applied Biosystems, USA). The LOD for each tissue in pg/µL is as follows: Nasal = 0.1, Lung = 0.02, Blood = 0.012.

### Quantitation of viable bacteria

Bullae were dissected post-mortem (both ears combined), homogenised and NTHi quantitated on spectinomycin-coated chocolate agar as described (12). For middle ear tissue, the LOD of CFU was calculated with a minimum of one colony in six 20μL spots using a 10^-1^ starting dilution, counted within a 20μL 10^-1^ diluted, multiplied by 50 to adjust to 1mL (1/6 = 0.166 CFU, 0.166 CFU × 10 = 1.66 CFU, 1.66 CFU × 50 = 83.33 CFU/mL [Log_10_ 1.92]. Samples below LOD were assigned half the LOD (Log_10_ 0.96) for statistical analysis.

### Statistical analysis

Proportional cell population data were analysed separately for each immune cell subset using generalised mixed-effects models fitted using *glmmTMB* (v1.1.10). The response variable was the proportion of each cell population, per tissue, per sample, modelled using a beta distribution with logit link to account for bounded proportional data. Fixed effects included Treatment, Tissue, and Timepoint (categorical), including the interaction terms. A random intercept for each animal (unique animal ID) accounted for within-subject correlation across tissues.

Model-derived estimated marginal means (EMM) were obtained using *emmeans* (v1.11.2) on the response scale. Pairwise contrasts between treatment groups were performed within each Tissue and Timepoint, using contrasts of EMM. The control for type I error arising from multiple comparisons across treatments, tissues, and timepoints, p-values were adjusted using the Benjamini-Hochberg false discovery rate correction (BH-FDR), applied within each Tissue across all Treatment comparisons and Timepoints for a given cell subset. FDR control was selected over more conservative family-wise error approaches to balance false-positive control with statistical power in the context of high-dimensional cytometry data. Statistical significance was defined as adjusted p < 0.05, with conventional thresholds applied for annotation.

All observations after data cleaning and transformation were included, and no experimental subjects were excluded. Extracted pairwise comparison results, including adjusted p-values and significance annotations, were collated across all cell subsets for downstream interpretation. All analyses were conducted in R (v4.4.0), and data visualisations were generated using *ggplot2*.

Wilcoxon rank-sum test was used to assess differences in middle ear tissue NTHi colonisation, using *ggsignif* (v0.6.4). For Hh distribution data, nasal tissue, lungs, and blood were collected from the same animal; statistical tests were performed separately within each tissue. Values below tissue-specific LODs (Nasal tissue 0.1µg/mL, Lungs 0.02µg/mL, Blood 0.012µg/mL) were assigned half the LOD (0.05, 0.01, 0.006 respectively), with adjusted values used for both plotting and inference. At each timepoint (1-96h) Hh vs Saline (independent animals; unpaired at each timepoint) were tested separately within each tissue using a two-way ANOVA-style linear model (Group × Timepoint), followed by estimated marginal means contrasts to assess group differences at each timepoint, with BH-FDR applied across the six timepoints per tissue. Boxplots show the median and interquartile range (IQR), with whiskers extending to 1.5× IQR and individual observations overlaid.

## Results

### *Haemophilus haemolyticus* is rapidly cleared from respiratory tissues after intranasal delivery indicating that persistent colonisation is not essential for prevention of NTHi OM

Immune responses following intranasal administration of Hh were investigated to understand the mechanism of action underlying its prevention of IAV-driven NTHi OM in mice. BALB/cJ mice were administered a single intranasal treatment comprising either saline (placebo), Hh in saline, or Pam2CSK4 in saline (Pam2CSK4 has been shown to prevent influenza infection (15)) (**Figure 1A**). At 24 hours post-treatment, mice were intranasally administered either saline or IAV, then either intranasal saline or NTHi at 96 hours (**Figure 1A**). This generated 12 study arms that enabled assessment of the cellular immune response to (i) Hh-only (ii) IAV-only (iii) NTHi-only (iv) IAV and NTHi, as well as impact of Hh or Pam2CSK4 treatment on the response to single IAV or NTHi challenge, and combined IAV-NTHi challenge (**Figure 1B**). Individual groups of mice were euthanised at 2, 6, 24, 48, 72, 96 and 144 hours after initial treatment (**Supplementary Table 1**). Nasal tissue, lungs and spleens were harvested and tissue-based cellular immunity assessed.

qPCR was used to measure Hh pharmacokinetics and biodistribution in nasal tissue, lungs and blood 1–96 hours post-administration (**Figure 1A**). Hh density decreased rapidly in nasal tissue, becoming undetectable 24–48 hours post-administration (**Figure 1C**). Intranasal delivery resulted in aspiration into the lungs, where lower Hh densities were detected compared to nasal tissues, and reached undetectable levels 6–24 hours post-administration (**Figure 1C**). Importantly, from a safety perspective, Hh was not detected systemically (in blood) after intranasal administration (**Figure 1C**). Confirming our previous studies in BALB/cARC mice, Hh treatment of BALB/cJ mice followed by intranasal challenge of IAV and NTHi reduced culturable NTHi by 141-fold in middle ear tissue (**Figure 1D**). 3 of 6 Hh-treated mice had completely undetectable levels of viable NTHi in their middle ear tissue, whereas all saline-treated mice developed middle ear NTHi counts >1x10^5^ CFU/mL at 144 hours (p=0.04; **Figure 1D**). In summary, Hh is rapidly cleared from respiratory tissues within 24–48 hours after intranasal administration, is not systemically distributed, and prevents NTHi infection of the middle ear upon challenge.

### High dimensional spectral flow cytometry of nasal tissue, lung and spleen identifies principal immune cell subsets

**Figure 2:**
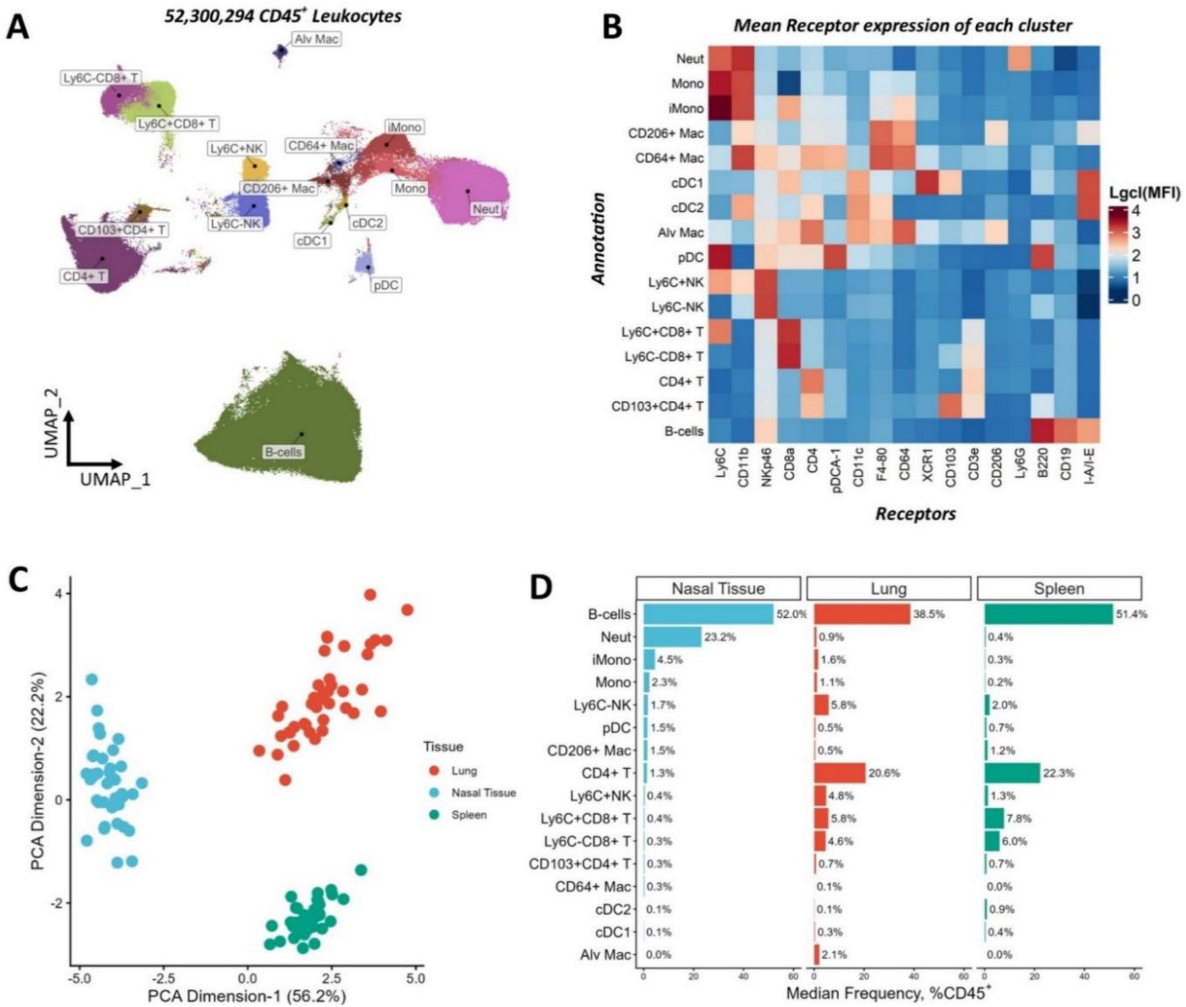
High Dimensional Analysis of immune cell subsets in BALB/cJ mice using full-spectrum flow cytometry. **(A)** 52,000,294 CD45^+^ Leukocytes were computationally analysed with the Louvain-based clustering algorithm PhenoGraph (implemented in R within Phenoannoy), resulting in 16 unique populations. **(B)** A heatmap demonstrating logicle-transformed median fluorescence intensities of lineage defining markers representing each annotated population. **(C)** Principal Component Analysis (PCA) was performed on immune subset frequencies in nasal tissues, lungs and spleens of intranasal saline treated mice 2–144 hours. **(D)** Median frequencies of each subset within each tissue analysed are presented.

Full-spectrum flow cytometry was used to determine innate and adaptive immune subsets elicited following intranasal Hh delivery (**Supplementary Table 2**). 52,300,294 CD45^+^ leukocytes were analysed from 702 individual organs, which underwent clustering with PhenoGraph (**Figure 2A**) and annotated for cellular identity according to phenotype (**Figure 2B**; **Supplementary Table 3**). 16 annotations were assigned including: Ly6G^+^CD11b^+^ neutrophils, CD64^+^ (classically-activated) macrophages, CD206^+^ (alternatively-activated) macrophages, CD11c^+^F4/80^+^CD64^+^ alveolar macrophages, XCR1^+^CD103^+^ classical dendritic cell 1 (cDC1), CD11b^+^ classical dendritic cell 2 (cDC2), pDCA-1^+^B220^+^ plasmacytoid dendritic cells (pDC), CD4^+^ T cells, CD103^+^CD4^+^ T cells, CD8^+^ T cells (Ly6C^+^, Ly6C^-^), NKp46^+^ NK cells (Ly6C^+^, Ly6C^-^), Ly6C^+^ classical monocytes, Ly6C^++^CD64^++^ inflammatory monocytes and CD19^+^B220^+^ B cells (**Supplementary Table 3**).

Principal component analysis (PCA) demonstrated global differences in immune cell frequencies between tissues at steady state (intranasal saline-treated mice at 2–144 hours; n=128; **Figures 2C, D**). B cells were the most frequent immune cell in all tissues, accounting for 38.5-52.0% of all CD45^+^ leukocytes (26). Neutrophils represented 23.2% of CD45^+^ leukocytes in nasal tissues (26), whereas they represented <1% of CD45^+^ leukocytes in lungs and spleens. Conversely, T cells were rare in nasal tissues, constituting 2.3% of CD45^+^ T cells. In spleens and lungs, CD4^+^ T cells represented 22.3% and 20.6% of CD45^+^ cells whereas CD8^+^ T cell subsets represented 13.8% and 10.4% of CD45^+^ cells, respectively (27, 28). CD103^+^CD4^+^ T cells were virtually absent in all tissues (29). NK cells were most frequent in lungs (30). Total cDCs represented <1% of CD45^+^ leukocytes in all tissues. In spleens, cDC2 were more frequent than cDC1, and CD206^+^ macrophages were more frequent than CD64^+^ macrophages in all tissues, demonstrating the Th2-bias reported in BALB/c mice (31). Alveolar macrophages were detected exclusively in the lungs. pDC represented ≤1.5% of all CD45^+^ leukocytes in each tissue (32). Classical and inflammatory monocytes represented <5% of CD45^+^ leukocytes in all of tissues, being most abundant in nasal tissue.

Our method provides baseline metrics of, and detected differences between, immune cell compositions prior to treatment and challenge.

### Intranasal *H. haemolyticus* results in the hyperacute infiltration of phagocytes and subsequent priming of lymphocytes in respiratory tissues

**Figure 3:**
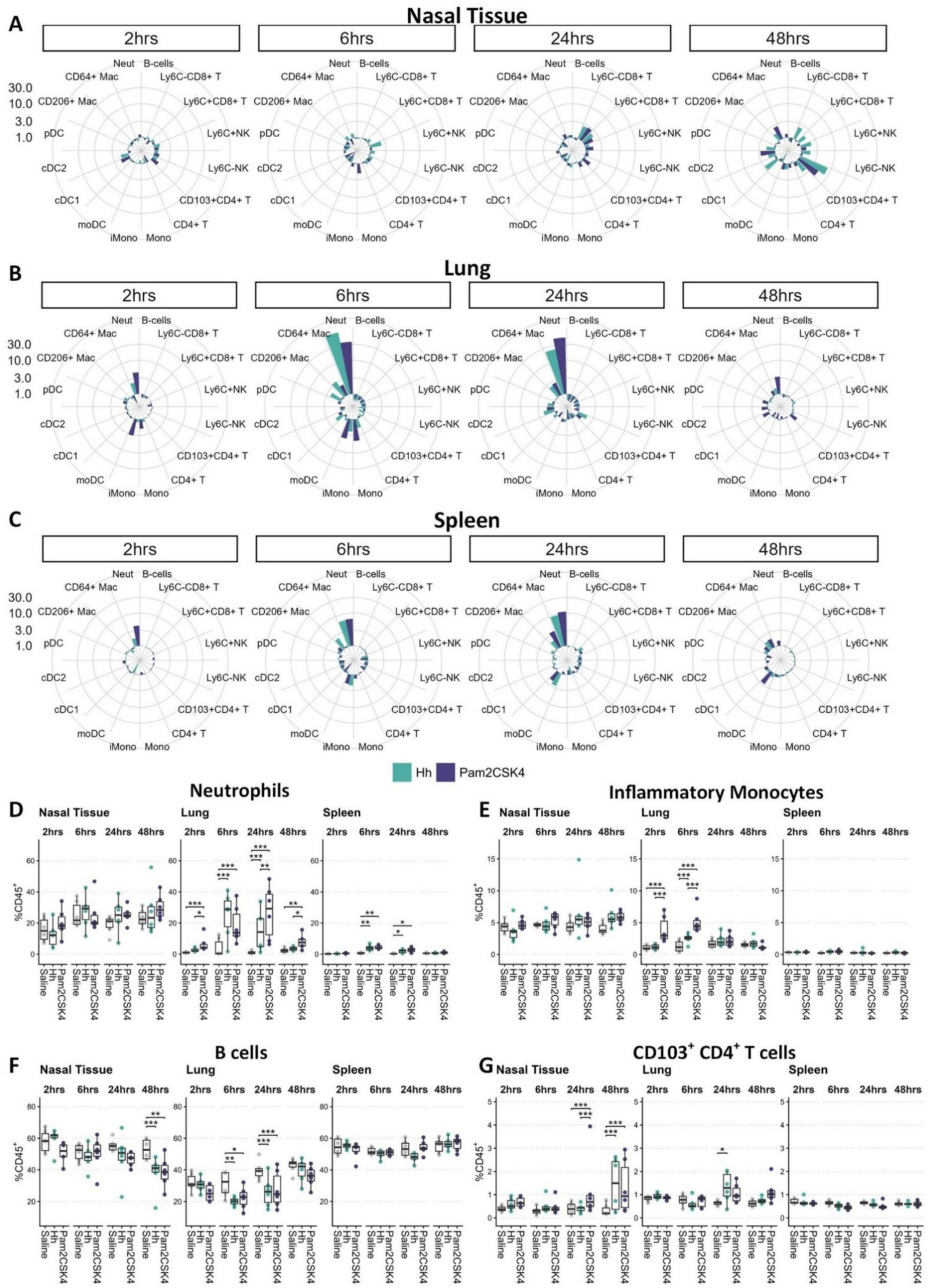
The acute immune response to *H. haemolyticus* and Pam2CSK4. Rose-Nightingale plots demonstrating fold change in immune cell subsets relative to saline within **(A)** nasal tissues, **(B)** lungs and **(C)** spleen at 2, 6, 24 and 48 hours after intranasal administration. Boxplots showing differences between frequencies of **(D)** Neutrophils, **(E)** Inflammatory monocytes, **(F)** B cells, **(G)** CD103^+^CD4^+^ T cells within each tissue. All collected data are shown; *n=6/Treatment/Timepoint.* (*, ** and *** represent p value <0.05, 0.01 and 0.001, respectively; Beta-regression with Logit Link and Benjamini-Hochberg correction for multiple comparisons).

Intranasal priming of immunity by bacterial PAMPs or live/killed pathogenic bacteria provides heterologous immunity to secondary viral or bacterial challenge. Less is known about immune priming effects by a human respiratory commensal such as Hh. We studied changes in immune cell subsets in lungs, nasal tissue and spleens harvested from Hh, saline or Pam2CSK4 treated mice at 2–144 hours after intranasal administration (Study arms 1-3; **Figure 1B**; **Figures 3A–C**). Most changes in immune cells, relative to saline controls, were observed within the first 48 hours after treatment (**Figures 3A–C**; **Supplementary Figure 1**) and coincided with Hh clearance kinetics (**Figure 1C**; **Supplementary Figure 1**).

Lungs exhibited the largest immune cell changes within the first 48 hours, driven by substantial increases in neutrophils to Hh and Pam2CSK4 treatment (**Figure 3B**). At 6 hours, Hh treated mice exhibited a median[IQR] lung neutrophil frequency of 28.8[17.6-34.2]%, compared to 0.39[0.17-7.84]% in saline-treated mice, corresponding with a median 74.1-fold higher response. This was higher than in Pam2CSK4 treated mice at the same timepoint (28.8[12.4-38.5]% vs 13.8[4.9-22.8]%; p<0.001; **Figure 3D**). The peak neutrophil response to Pam2CSK4 was slower (**Figure 3B**); reaching a median 47.3-fold increase from saline at 24 hours and was higher than in Hh treated mice at this timepoint (29.2[12.4-38.5]% vs 14.2[4.9-22.8]%; p<0.0001; **Figure 3D**). In spleen, both Hh and Pam2CSK4 treated mice exhibited neutrophil increases (**Figure 3C**) peaking at a median 6.4-fold and 6.7-fold higher frequencies than in saline-treated mice at 6 hours (3.8[2.6-4.8]% vs 3.9[3.6-5.2]% vs 0.6[0.2-1.0]%, respectively, p<0.0001; **Figure 3D**).

Inflammatory monocytes (iMono) were 3.9- and 2.3-fold higher in the lungs of Pam2CSK4 and Hh treated mice compared to saline at 6 hours, respectively (**Figure 3B**). In the lungs at this timepoint, Pam2CSK4 induced the highest frequencies of iMono, followed by Hh then saline (4.4[3.9-5.3]% vs 2.6[2.4-2.8]% vs 1.1[0.6-2.0]%, respectively; p<0.0001; **Figure 3E**).

B-cells in the lungs were 1.6- and 1.4-fold lower in Hh and Pam2CSK4 treated mice compared to saline controls at 6 hours, respectively (**Figure 3B**). Frequencies of B cells were lowest in Hh followed by Pam2CSK4 and then saline treated mice (20.1[18.5-21.5]% vs 22.8[18.3-25.6]% vs 32.5[25.7-38.3]%, respectively; **Figure 3F**) at 6 hours, suggesting potential egress from the tissue. The difference was similar in the lungs at 24 hours and no longer observed at 48 hours. At 48 hours, B-cells in nasal tissues were 1.3- and 1.4- fold lower in Hh and Pam2CSK4 treated mice compared to saline controls (41.1[38.6-42.9]% vs 38.7[34.3-42.9]% vs 52.7[47.0-58.8]%; **Figure 3F**).

Infection re-programs tissue T cells to differentiate toward the residency lineage and upregulate CD103 expression. At 24 hours, CD103^+^CD4^+^ T cells in nasal tissues were higher in Pam2CSK4 compared to Hh or saline treated mice (**Figure 3A**). This corresponded to nasal tissue frequencies of 0.69[0.5-1.0]% vs 0.44[0.3-0.5]% vs 0.38[0.2-0.6]% for Pam2CSK4, Hh and saline, respectively (p<0.001; **Figure 3G**). At 48 hours, CD103^+^CD4^+^ T cells were 7.8- and 4.9-fold higher in nasal tissue of Hh and Pam2CSK4 treated mice compared to saline controls (1.5[0.4-2.4]% vs 0.94[0.4-2.2]% vs 0.2[0.2-0.4]%, respectively; p<0.0001; **Figure 3G**).

All other cell subsets exhibited minimal differences (**Supplementary Figure 2**). These findings were also observed when analysing cell counts (**Supplementary Figures 5A–D**). In summary, Hh and Pam2CSK4 treatments resulted in immune changes that were temporally distinct, respiratory tissue-specific and involved both innate and adaptive immune subsets, within 48 hours of intranasal administration.

### Mice treated with *H. haemolyticus* have increased nasal CD103^+^CD4^+^ T cells and reduced natural killer cells following IAV infection

**Figure 4:**
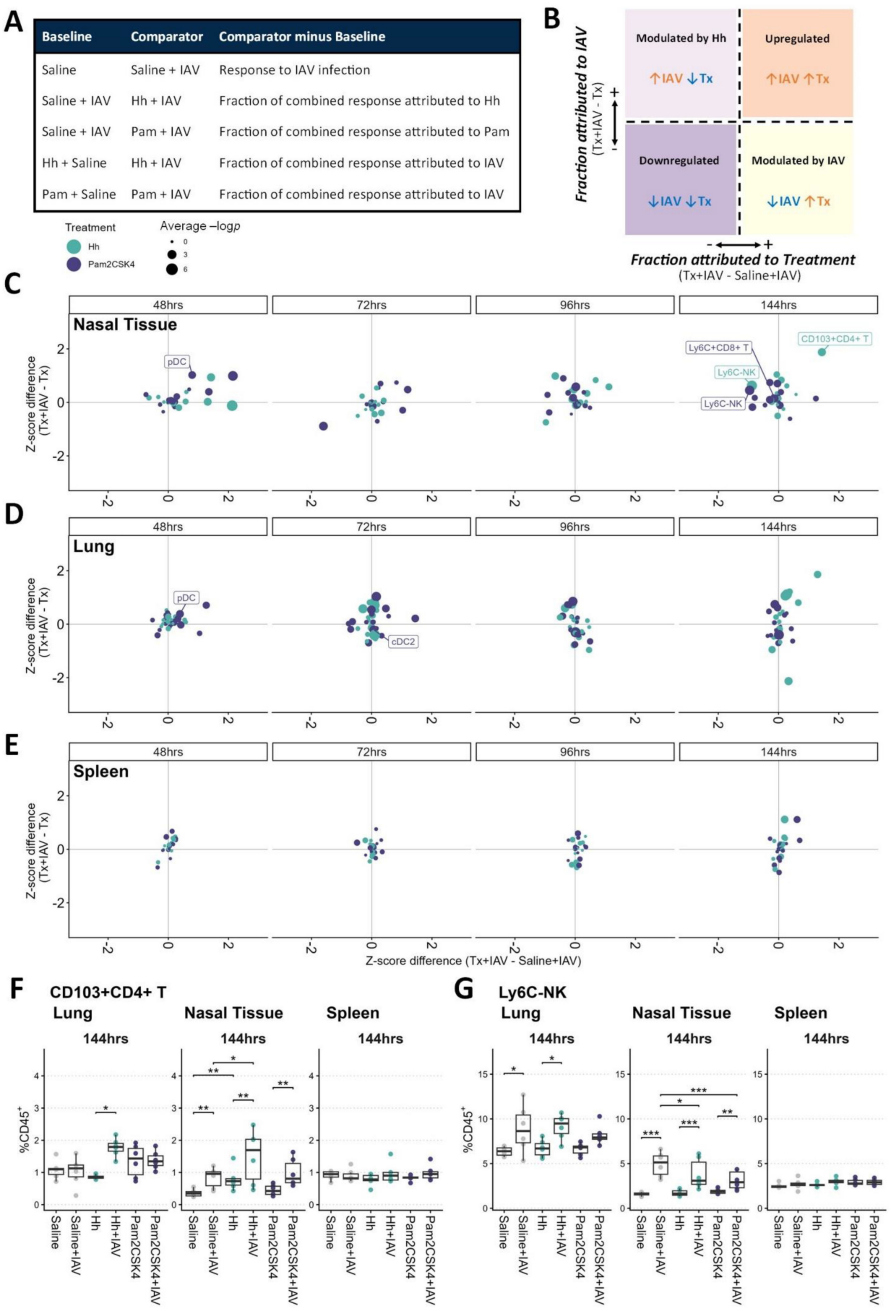
Effects of IAV infection and treatment on the cellular immune response. **(A)** A table summarising baseline and comparator groups to delineate single and combined effects of treatment and IAV infection. **(B)** A representative plot illustrates the screening of potentially modulatory, up-regulatory and down-regulatory effects of one stimulus with respect to the other, when both are combined. Contributions of each stimulus to the combined response (Treatment+IAV) are visualised for **(C)** nasal tissues, **(D)** lungs, and **(E)** spleens at 48, 72, 96 and 144 hours. Labels for cellular subsets are presented on plots if the combined response was significantly different to both individual stimuli (p < 0.05). p values (transformed with -log) were averaged for both comparisons (combined versus treatment only and combined versus IAV only) and mapped to the size of each point, to enhance visualisation. Individual responses for **(F)** CD103^+^CD4^+^ T cells and **(G)** Ly6C^-^ NK cells are visualised for each group. Boxplots of all cellular populations are shown in **Supplementary Figure 3**. (*, ** and *** represent p value <0.05, 0.01 and 0.001, respectively; Beta-regression with Logit Link and Benjamini-Hochberg correction for multiple comparisons). n=6/Treatment/Timepoint.

To investigate cellular immune factors associated with Hh protection to IAV, mice treated with Hh, Pam2CSK4 or saline were intranasally challenged with IAV or saline 24 hours after treatment. Six study arms followed until 144 hours post-treatment (arms 1-6; **Figure 1B**) facilitated the analysis of singular or combined effects of treatment and IAV infection (**Figure 4A**). As individual administration of IAV and Hh induce distinct cellular immune changes, it was important to factor this in the analysis of Hh+IAV responses, so that additive or modulatory effects could be differentiated from those induced by singular stimulus.

IAV-only and Hh-only responses were subtracted from combined Hh+IAV responses, enabling screening of IAV responses that were potentially modulated, upregulated or downregulated with Hh treatment (**Figure 4B**). Though not the focus of this work, the potential effect of IAV infection on modulating the immunostimulatory effects of Hh could also be evaluated (**Figure 4B**). The same analysis was performed for IAV infection with Pam2CSK4 treatment, as a comparator.

Major modulatory responses were observed in nasal tissues, specifically at 144 hours (**Figure 4C**). Hh treatment and IAV infection were both associated with upregulation of CD103^+^CD4^+^ T cells (**Figure 4C**). However, Hh treatment was associated with lower Ly6C^-^ NK cells relative to IAV infection at 144 hours in nasal tissues (**Figure 4C**). Similarly, Pam2CSK4 differentially reduced Ly6C^+^ NK cells and Ly6C^+^CD8^+^ T cells in the nasal tissues (**Figure 4C**) relative to IAV infection. Some smaller magnitude effects on pDC and cDC2 subsets were observed in nasal tissues and lungs with Pam2CSK4 treatment at 48 and 72 hours (**Figure 4D**). There were no significant changes observed in the spleen (**Figure 4E**).

When focusing on individual responses between treatments, CD103^+^CD4^+^ T cells in nasal tissue were higher in the Hh+IAV group compared to Saline+IAV (1.70[0.8-2.0]% vs 0.96[0.6-1.0]%, p = 0.02) at 144 hours (**Figure 4F**; **Supplementary Figures 3**, **5E**). Ly6C^-^ NK cells were lower in nasal tissue of Hh+IAV compared to Saline+IAV groups at 144 hours (5.2[3.8-5.9]% vs 3.1[2.7-5.2]%, p = 0.04; **Figure 4G**; **Supplementary Figures 3**, **5F**).

Overall, Hh and Pam2CSK4 treatment was associated with down-modulation of cytolytic immune cell subsets following IAV challenge. However, Hh was unique from Pam2CSK4 in driving an increase in CD103^+^CD4^+^ T cells in nasal tissues following IAV infection at 144 hours.

### The host response to nontypeable *H. influenzae* is immunologically attenuated compared to *H. haemolyticus*

Before investigating the cellular features associated with Hh-mediated protection against NTHi infection, we first sought to understand the host response to intranasal NTHi challenge.

**Figure 5:**
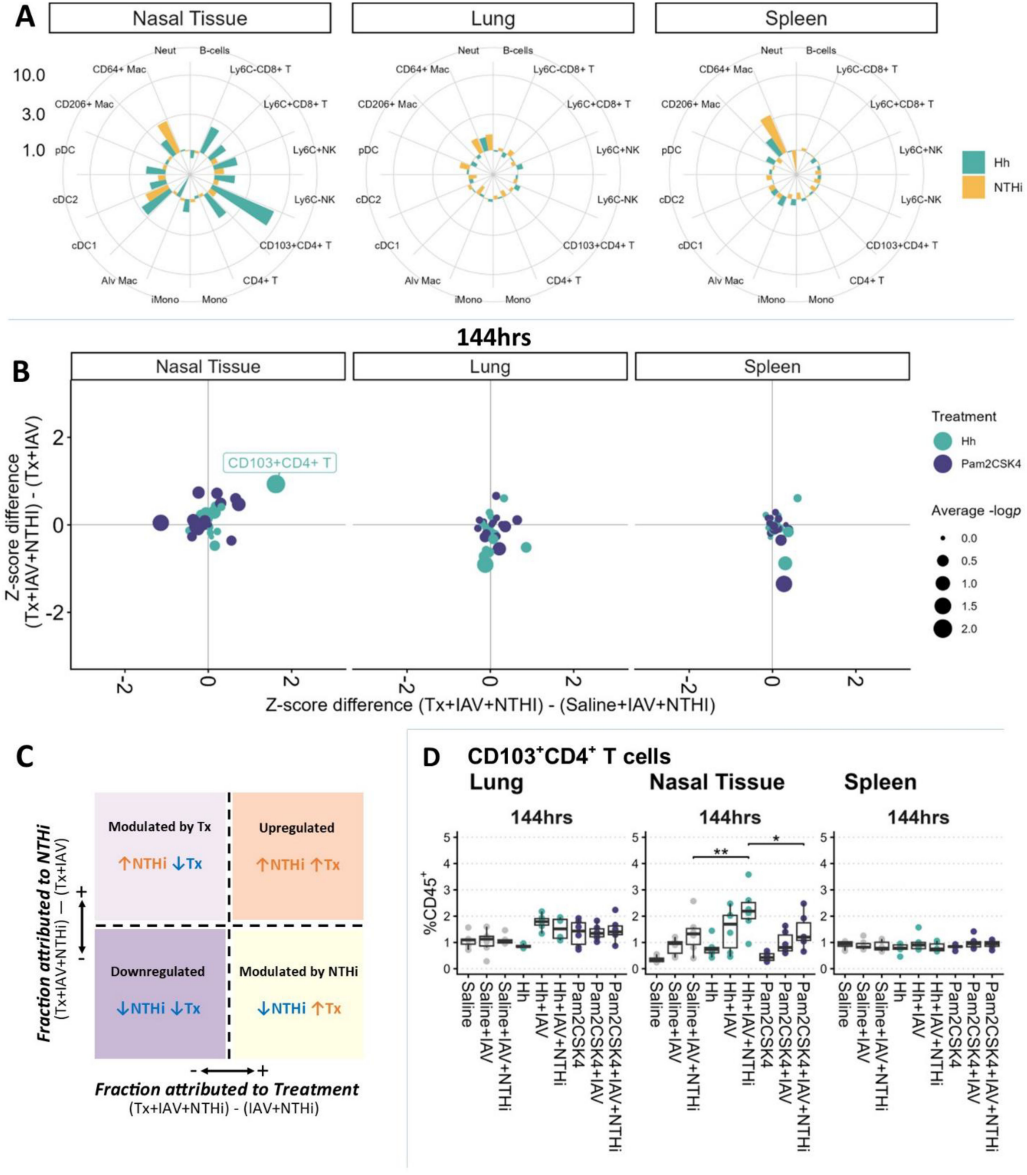
The cellular immune response to NTHi or Hh 48 hours after intranasal administration. **(A)** Cellular immune changes 48 hours after intranasal administration of Hh or NTHi in Nasal Tissue, Lung and Spleens of BALB/cJ mice are shown as fold change relative to respective saline controls. **(B, C)** The relative changes induced by NTHi challenge (ΔNTHi) and Treatment (ΔTreatment) are represented in biaxial plots to map discordant or shared responses. Boxplots showing differences between frequencies of **(D)** CD103+CD4+ T cells within each tissue at 144 hours. Boxplots of all cellular populations are shown in **Supplementary Figure 4**. (* and ** represent p value <0.05 and 0.01, respectively; Beta-regression with Logit Link and Benjamini-Hochberg correction for multiple comparisons). n=6/Treatment/Timepoint.

With the samples available, immune responses were only comparable at 48 hours for NTHi solo challenge (study Arm 9; **Figure 1B**) and Hh treatment (study arm 1; **Figure 1B**). The largest immune changes at 48 hours post-administration were observed in nasal tissues (**Figure 5A**), where Hh resulted in positive median fold changes in CD103^+^CD4^+^ T cells (7.08), cDC1 (2.80), Ly6C^-^CD8^+^ T cells (2.36), Ly6C^+^CD8^+^T cells (1.78), Ly6C^+^ NK cells (2.08), Ly6C^-^ NK cells (1.87), pDC (1.81), CD64^+^ Macrophages (1.72) and cDC2 (1.66) (**Figure 5A**). In contrast, NTHi induced fewer changes in nasal tissues, mainly in the upregulation of CD64^+^ Macrophages (2.84) and cDC1 (2.80). CD64^+^ macrophages in the spleen were increased for both Hh and NTHi treated mice, and more so following NTHi challenge (3.49; **Figure 5A**).

These data demonstrate that NTHi is immune evasive in the absence of priming stimuli, whereas Hh has immunostimulatory properties.

### *H. Haemolyticus* primes CD4^+^ T cells following intranasal NTHi challenge

Evaluation of the impact of Hh treatment on the immune response to NTHi in the full IAV-NTHi OM model (including IAV challenge at 96 hours; Study Arms 10-12; **Figure 1B**) revealed that Hh-treatment was associated with upregulation of CD103^+^CD4^+^ T cells in nasal tissues, above that afforded by NTHi administration alone (**Figures 5B, C**). This was confirmed when visualising individual CD103^+^CD4^+^ T cell frequencies, demonstrating that CD103^+^CD4^+^ T cells were higher in Hh+IAV+NTHi mice compared to the saline+IAV+NTHi control group (2.20[1.9-2.5]% vs 1.3[0.9-1.5]%, *p* = 0.005; **Figure 5D**; **Supplementary Figures 4**, **5G**). Minimal differences in CD103^+^CD4^+^ frequencies were observed with Pam2CSK4 treatment, compared to saline.

## Discussion

NTHi is the leading pathogen causing chronic and recurrent OM (8, 9). Lower respiratory tract infections with NTHi are associated with asthma exacerbations in children (33), pneumonia (10), and acute exacerbations of chronic obstructive pulmonary disease requiring hospitalisation (34). Despite its clinical significance, no licensed vaccine exists for the prevention of NTHi infection (35, 36), underscoring the urgent need for novel prophylactic strategies to treat NTHi-OM. Given the synergistic role of respiratory viruses such as influenza in facilitating secondary NTHi infections, interventions that confer broad-spectrum, mucosal immunity are of significant value. In this study, we demonstrate that Hh primed innate and adaptive responses that were not observed with a synthetic PAMP, offering unique mechanisms by which a live commensal bacterium enhances host resilience to viral and bacterial infections.

Hh colonises the nasopharynx of humans and is closely related to NTHi (37). In our mouse model, Hh was cleared from nasal and lung tissue 48 hours post-administration, demonstrating respiratory tract delivery but without persistent colonisation. These findings have several implications. First, the mouse model reveals protective mechanisms beyond persistent competitive colonisation between Hh and NTHi, a mechanism shown to occur *in vitro* with human respiratory cell lines (38, 39). Second, we observed a substantial innate immune response in lungs within 24 hours concordant with distribution of Hh in the lungs in our mouse model. This contrasts with other mouse studies (19, 40), likely due to biodistribution kinetics that differ from Hh. The intranasally-delivered, live-attenuated *Bordetella pertussis* strain BPZE1 is also distributed to the lungs (41), though important to note that unlike Hh, BPZE1 persistently colonises host mice. Distribution of bacteria may differ in the human setting as mice are physiologically limited to breathing through the nose (42), increasing the likelihood of passive aspiration. Despite these considerations, our model facilitated assessment of immune dynamics following transient presence of Hh and subsequent heterologous pathogen challenge, in the absence of persistent colonisation, providing first insights into Hh-driven immune priming.

Hh treatment was characterised by rapid and high influx of neutrophils into lungs, concordant with our previous findings that Hh stimulates the release of nasal KC (a functional ortholog of human CXCL1) in mice (unpublished data) and IL-8 from cultures of human respiratory epithelial cells (38), which are potent neutrophil chemo-attractants. Our findings support similar observations following intranasal delivery of commensal *Prevotella* and *Corynebacterium* spp. in mice (43, 44). In the former, *Prevotella melaninogenica* primed neutrophils to express TNFα via activation of TLR2, required for protection against *Streptococcus pneumoniae* challenge (43) and neutrophil-derived anti-inflammatory IL-10 attenuated adverse inflammation associated with *S. pneumoniae* infection (43). Similarly, the BPZE1 vaccine that is protective against IAV (4, 45) and *S. pneumoniae* infection (46) decreases inflammatory cytokine levels in the respiratory tract to prevent the damaging cytokine storm upon challenge (4). We have shown that Hh reduces inflammatory cytokine responses in the lungs of mice in the IAV-NTHi OM model, in a dose-dependent manner (13). Thus, we propose that Hh-mediated protection and innate priming effects, while measurable primarily in the lungs of mice, extend beyond the lower respiratory tract to provide reduced inflammation and host susceptibility to IAV-NTHi OM.

Hh differs to Pam2CSK4 in that it is a live whole cell bacteria harbouring multiple PAMPs and shares conserved antigens with NTHi (25). In contrast, Pam2CSK4 is a synthetic agonist that specifically activates the TLR2/6 pathway. Incidentally, a structurally similar Pam2Cys derivative has also been shown to prevent IAV infection (15, 19) like Hh (13) further highlighting the importance of innate responses against viral and bacterial pathogens. NTHi outer membrane protein (OMP) P6 is known to activate TLR2 via terminal Pam3Cys motifs (47). Hh expresses P6 protein (with slight (4/153) amino acid differences to NTHi P6) (37), so it is possible that a portion of the Hh immune response is mediated via TLR2. Additionally, Hh express *lic* genes (with sequence homology to those of NTHi) (48) encoding phosphorylcholine kinases required for lipo-oligosaccharide synthesis, that are ligands of TLR4 (49). Comparative genomic analyses between our NTHi and Hh strains are the subject of ongoing investigation. Molecular differences may explain immunostimulatory versus immune-evasive properties of Hh and NTHi, respectively.

Beyond innate immune activation, we demonstrated evidence of adaptive immune priming with Hh, not observed with Pam2CSK4 treatment. Although the timepoints in this study were too early to evaluate matured antibody and B cell responses, we observed elevated CD103^+^CD4^+^ T cells in nasal tissues after Hh treatment, that successively increased following IAV and NTHi challenges. While we did not identify the function(s) of these T cells in our model, CD103^+^CD4^+^ T cells could mediate protection in several ways. First, increased abundance of CD4^+^ T cells may enhance tissue immune surveillance, which is important given our finding that NTHi cellular immunogenicity is low in our model and that NTHi has well established immune evasion mechanisms. Linked with enhanced surveillance, CD103^+^CD4^+^ T cells can induce bystander protection, as has been observed with *Bordetella pertussis* priming and protection to *Klebsiella pneumoniae (*50). Finally, CD103^+^CD4^+^ T cells may provide cross-protection to similarly related pathogens, a possibility given the sequence homology between Hh and NTHi (51). In this regard, it would be necessary to conduct future experiments defining the functional contribution(s) of Hh-induced CD103^+^CD4^+^ T cells, and their specificity, to differentiate cross-protection versus heterologous protection to NTHi.

Given the impracticality of sampling nasal and lung tissue in human trials, alternative, minimally invasive methods such as nasal swabs and peripheral blood collection are essential for immunological monitoring. Nasopharyngeal swabs offer a valuable and reliable tool for assessing local mucosal immune responses to respiratory vaccines and infections (52, 53). Our findings inform inclusion of neutrophils and CD103^+^CD4^+^ T cells as markers of cellular mucosal immunogenicity in future clinical trials of intranasal Hh vaccination, alongside nasal aspirates for cytokine quantification, as potential biomarkers of immune activation.

There are several limitations in this study. Due to experimental requirements, clinical and immunological endpoints could not be measured in the same animal, precluding paired immune correlates analyses. Potential sex-based differences could not be evaluated due to limited sample sizes. In addition, we could not conclude mechanistic contributions of Hh-induced neutrophils and CD103^+^CD4^+^ T cells to protection, requiring future experiments to examine the effect of neutrophil and/or CD103^+^CD4^+^ T cell depletion. Such experiments could involve specific depletion of these cell populations with monoclonal antibodies, as has been previously demonstrated in other studies (54, 55).

This study highlights the capacity of Hh to stimulate heterologous immunity involving early priming of innate cells and T cells, distinct from those observed with synthetic stimulants and similar to live attenuated bacterial nasal vaccines, such as BPZE1 (4, 45, 46). Together with evidence of Hh-mediated protection and enhanced efficacy with repeated dosing (20), these findings underscore the potential of an intranasal vaccine comprised of a live commensal bacteria to provide a needle-free strategy with broad protection, as a viable approach to address the unmet need for NTHi and respiratory disease prevention.

## Data Availability

The raw data supporting the conclusions of this article will be made available by the authors, without undue reservation.

## Footnotes

1 https://github.com/immunedynamics/spectre.

2 https://github.com/stuchly/Rphenoannoy.

3 https://github.com/jlmelville/uwot.

4 https://github.com/jlmelville/rcpphnsw.
