# Supplementary material for: Mucosal immune cell priming by intranasally delivered Haemophilus haemolyticus is associated with heterologous protection against influenza and nontypeable Haemophilus influenzae
Source: Front Immunol. 2026 Jul 20;17:1855464. doi: 10.3389/fimmu.2026.1855464 (PMC13429789; doi:10.3389/fimmu.2026.1855464)
Supplement: Supplementary file 1 [file Table1.docx]

**Supplementary Information**

**Mucosal immune cell priming by intranasally delivered *Haemophilus haemolyticus* is associated with heterologous protection against Influenza and nontypeable *Haemophilus influenzae***

Jack S. Pepper^1^, Caitlyn M. Granland^1^, Sharon L. Clark^1^, Ruth B. Thornton^2^, Josephine Bayliss^1^, M. Z. Edison Foo^1^, Wesley Billingham^3^, Naomi Scott^1^, Alma Fulurija^1,2^, Deborah H. Strickland^1,4-6^, Selma P. Wiertsema^7^, Peter C. Richmond^1,8,9^, Elke J. Seppanen^1,4^, Lea-Ann S. Kirkham^1,4^ and M. Christian Tjiam^1,2,*^

^1^Wesfarmers Centre of Vaccines and Infectious Diseases, The Kids Research Institute Australia, Perth, Australia

^2^School of Biomedical Sciences, The University of Western Australia, Perth, Australia

^3^The Kids Research Institute Australia, Perth, Australia

^4^Centre for Child Health Research, The University of Western Australia, Perth, Australia

^5^Medical, Molecular and Forensic Sciences, Murdoch University, Perth, Western Australia, Australia

^6^Wal-yan Respiratory Research Centre, The Kids Research Institute Australia, Perth, Australia.

^7^BioscienceConnect, Utrecht, The Netherlands.

^8^Discipline of Paediatrics, Medical School, The University of Western Australia, Perth, Australia

^9^Immunology and General Paediatrics Departments, Perth Children’s Hospital, Perth, Australia

**Supplementary Figure 1.** Fold change of immune cell subsets in Hh or Pam2CSK4 treated mice at 2-144hrs within the study timeline.


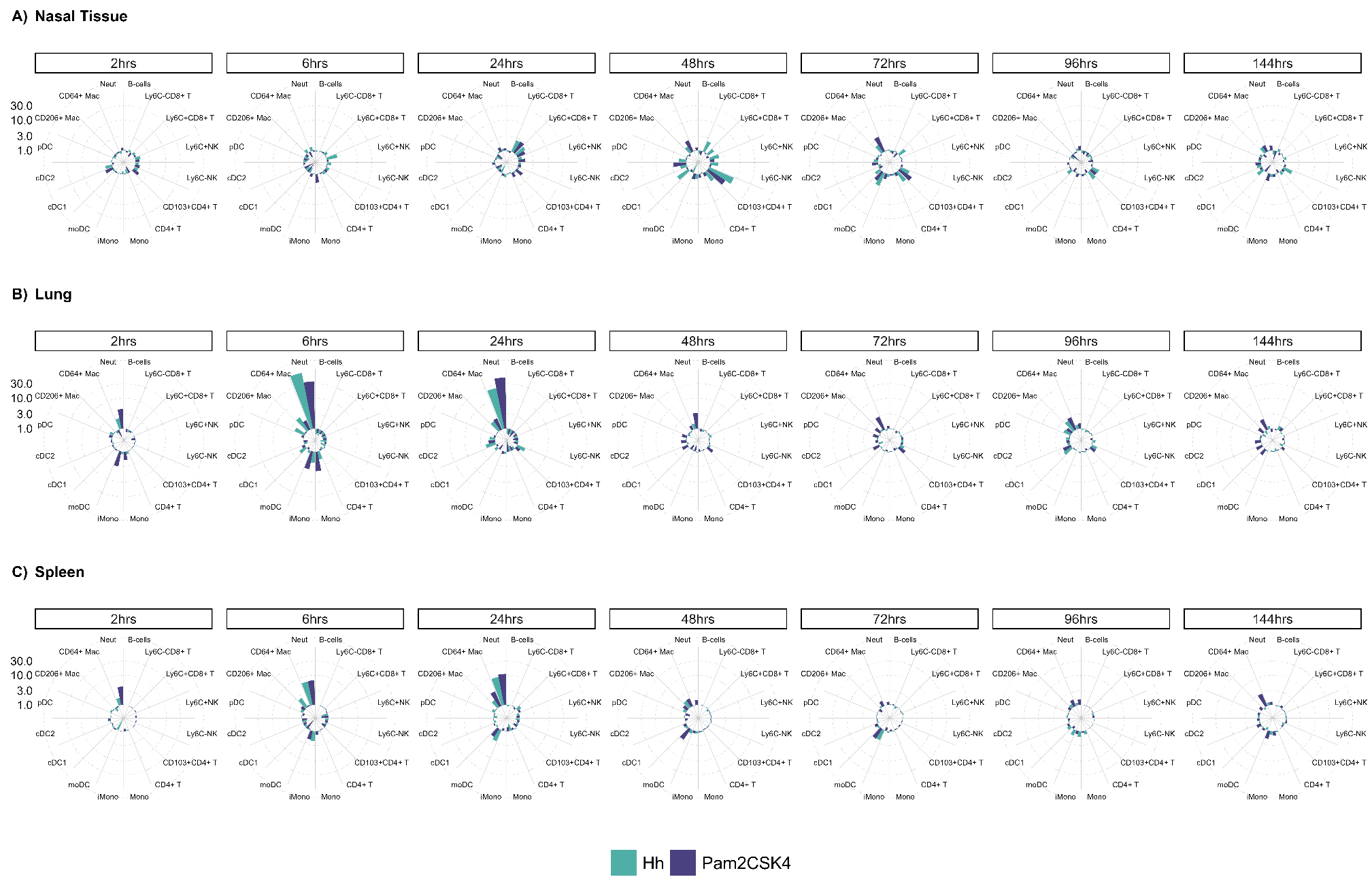


**Supplementary Figure 1**. Rose plots showing the fold change of immune cell subsets in Hh or Pam2CSK4 treated mice at 2-144hrs within the study timeline. Fold change was calculated from mean population frequency of treated mice against the mean population of mice receiving Placebo. Neut, Neutrophils; CD64+ Mac, CD64+ M1-like Macrophages; CD206+ Mac, CD206+ M2-like Macrophages; Alv Mac, Alveolar Macrophage; cDC1, Classical Dendritic Cell 1; cDC2, Classical Dendritic Cell 2; pDC, Plasmacytoid Dendritic Cell; CD4+ T, CD4+ T-cells; CD103+CD4+ T, CD103+CD4+ T-cells; Ly6C+CD8+ T, Ly6C+CD8+ T-cells; Ly6C-CD8+ T, Ly6C-CD8+ T-cells; Ly6C+ NK, Ly6C+ NK cells; Ly6C- NK, Ly6C- NK cells; Mono, Classical Monocytes; iMono, Inflammatory Monocytes.

**Supplementary Figure 2**
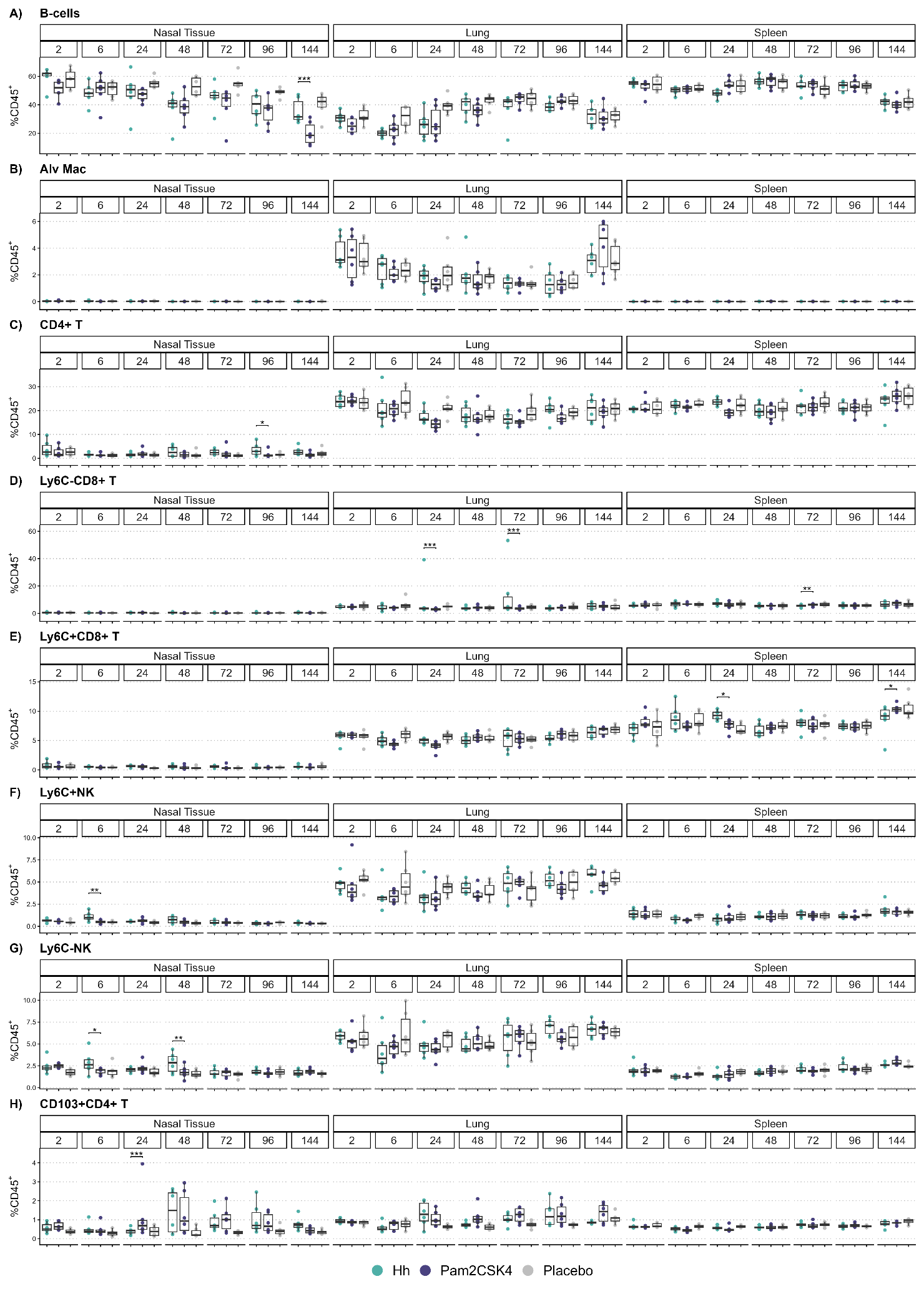

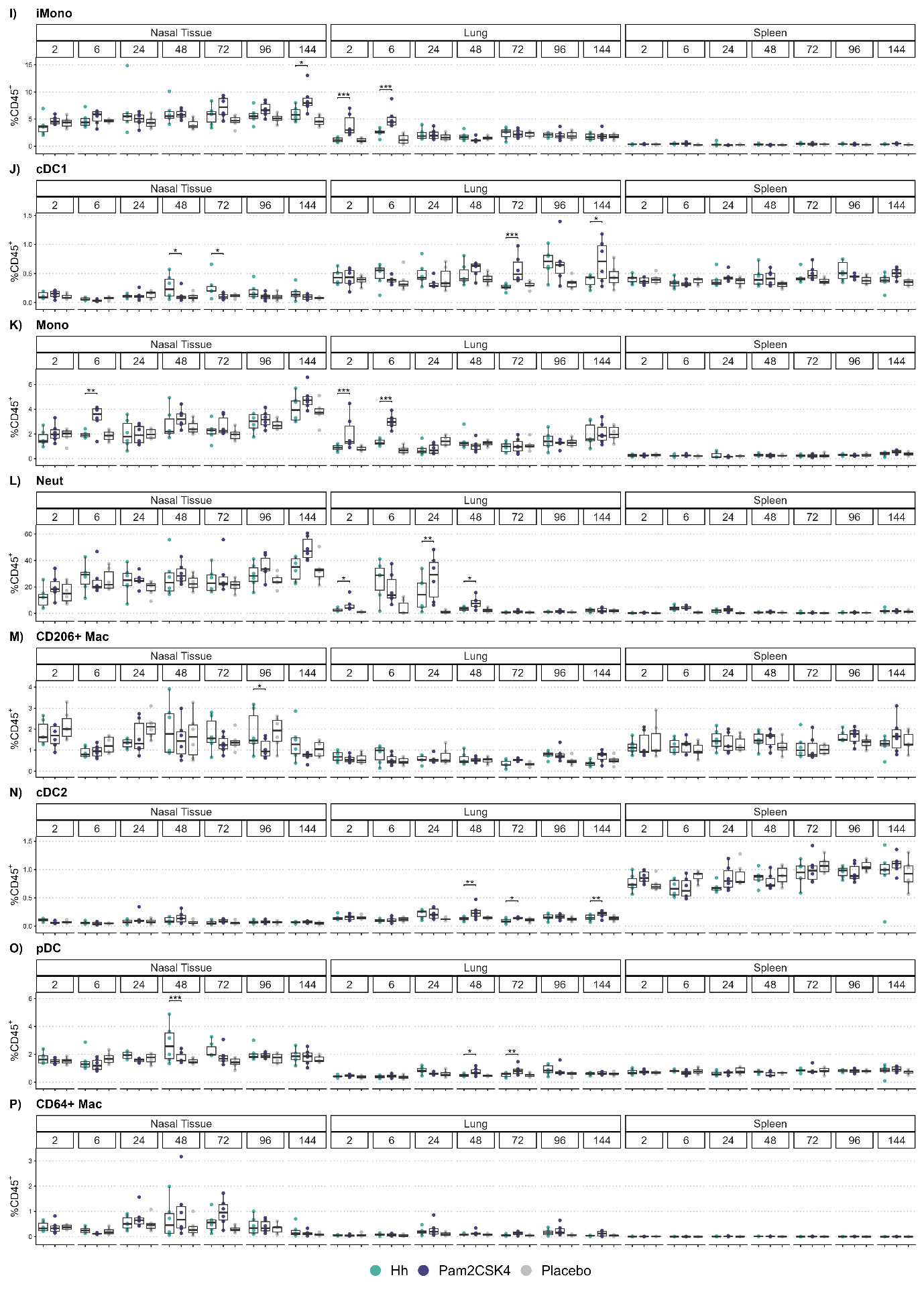


**Supplementary Figure 2**. Boxplots showing the difference between population frequencies of all populations annotated within this dataset across all pre-treatment conditions in the absence of IAV and NTHi challenge, tissues, and timepoints. N=6/treatment/tissue/timepoint. (*, ** and *** represent p value <0.05, 0.01 and 0.001, respectively). Neut, Neutrophils; CD64+ Mac, CD64+ M1-like Macrophages; CD206+ Mac, CD206+ M2-like Macrophages; Alv Mac, Alveolar Macrophage; cDC1, Classical Dendritic Cell 1; cDC2, Classical Dendritic Cell 2; pDC, Plasmacytoid Dendritic Cell; CD4+ T, CD4+ T-cells; CD103+CD4+ T, CD103+CD4+ T-cells; Ly6C+CD8+ T, Ly6C+CD8+ T-cells; Ly6C-CD8+ T, Ly6C-CD8+ T-cells; Ly6C+ NK, Ly6C+ NK cells; Ly6C- NK, Ly6C- NK cells; Mono, Classical Monocytes; iMono, Inflammatory Monocytes.

**Supplementary Figure 3**
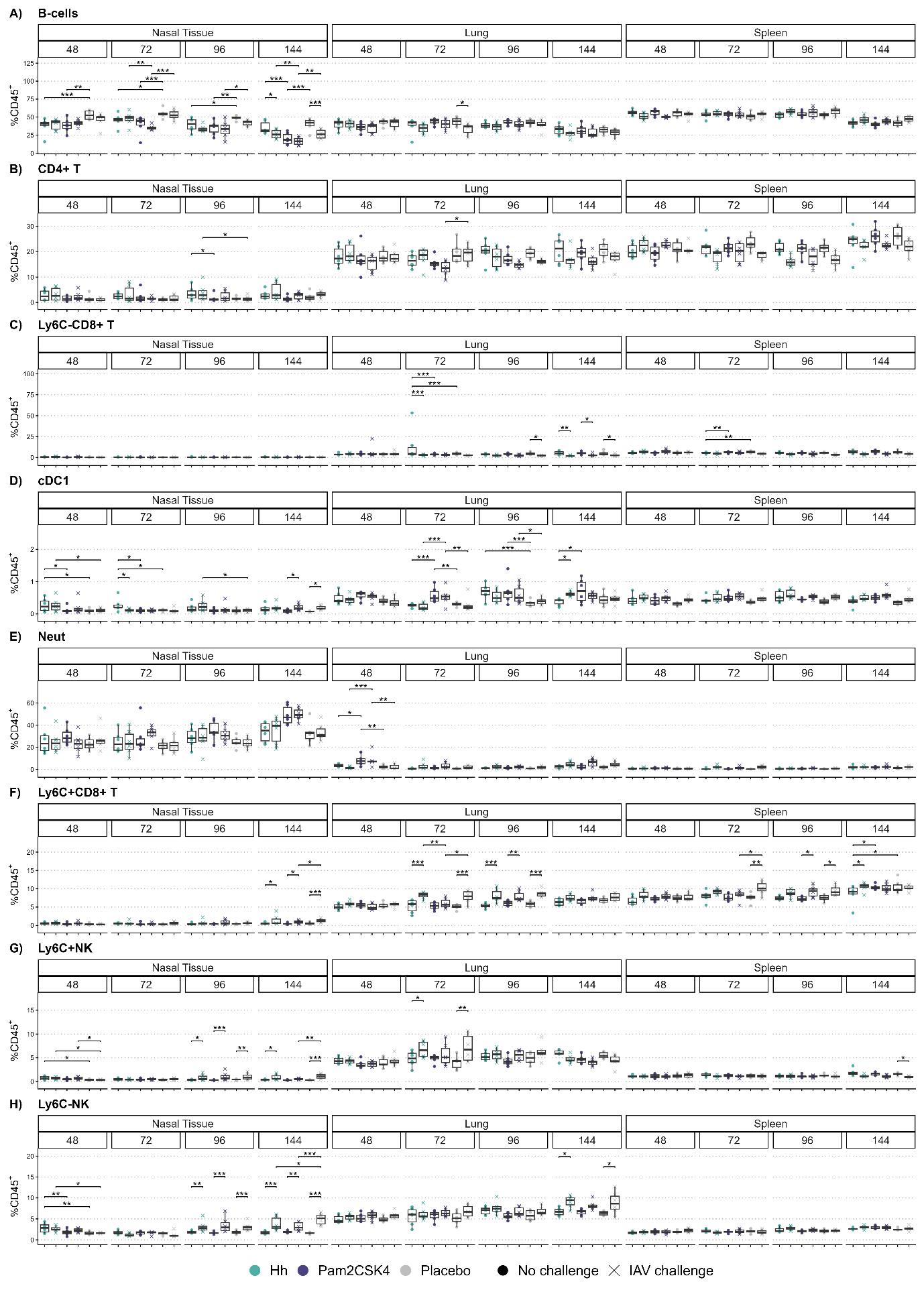

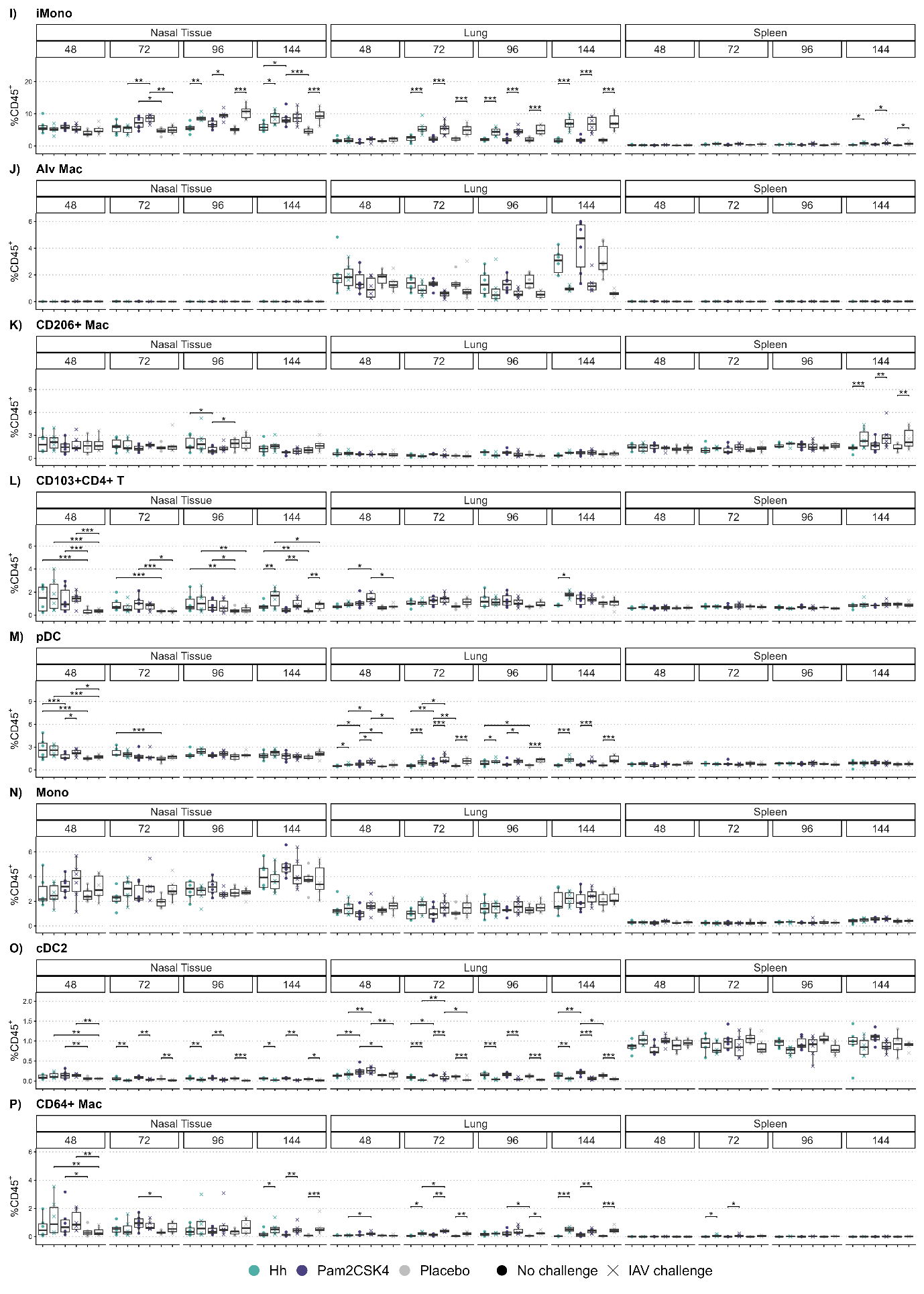


**Supplementary Figure 3**. Boxplots showing the difference between population frequencies of all populations annotated within this dataset across all pre-treatment conditions in the absence of NTHi challenge, and/or IAV challenge, tissues, and timepoints. N=6/treatment/tissue/timepoint. (*, ** and *** represent p value <0.05, 0.01 and 0.001, respectively). Neut, Neutrophils; CD64+ Mac, CD64+ M1-like Macrophages; CD206+ Mac, CD206+ M2-like Macrophages; Alv Mac, Alveolar Macrophage; cDC1, Classical Dendritic Cell 1; cDC2, Classical Dendritic Cell 2; pDC, Plasmacytoid Dendritic Cell; CD4+ T, CD4+ T-cells; CD103+CD4+ T, CD103+CD4+ T-cells; Ly6C+CD8+ T, Ly6C+CD8+ T-cells; Ly6C-CD8+ T, Ly6C-CD8+ T-cells; Ly6C+ NK, Ly6C+ NK cells; Ly6C- NK, Ly6C- NK cells; Mono, Classical Monocytes; iMono, Inflammatory Monocytes.

**Supplementary Figure 4**


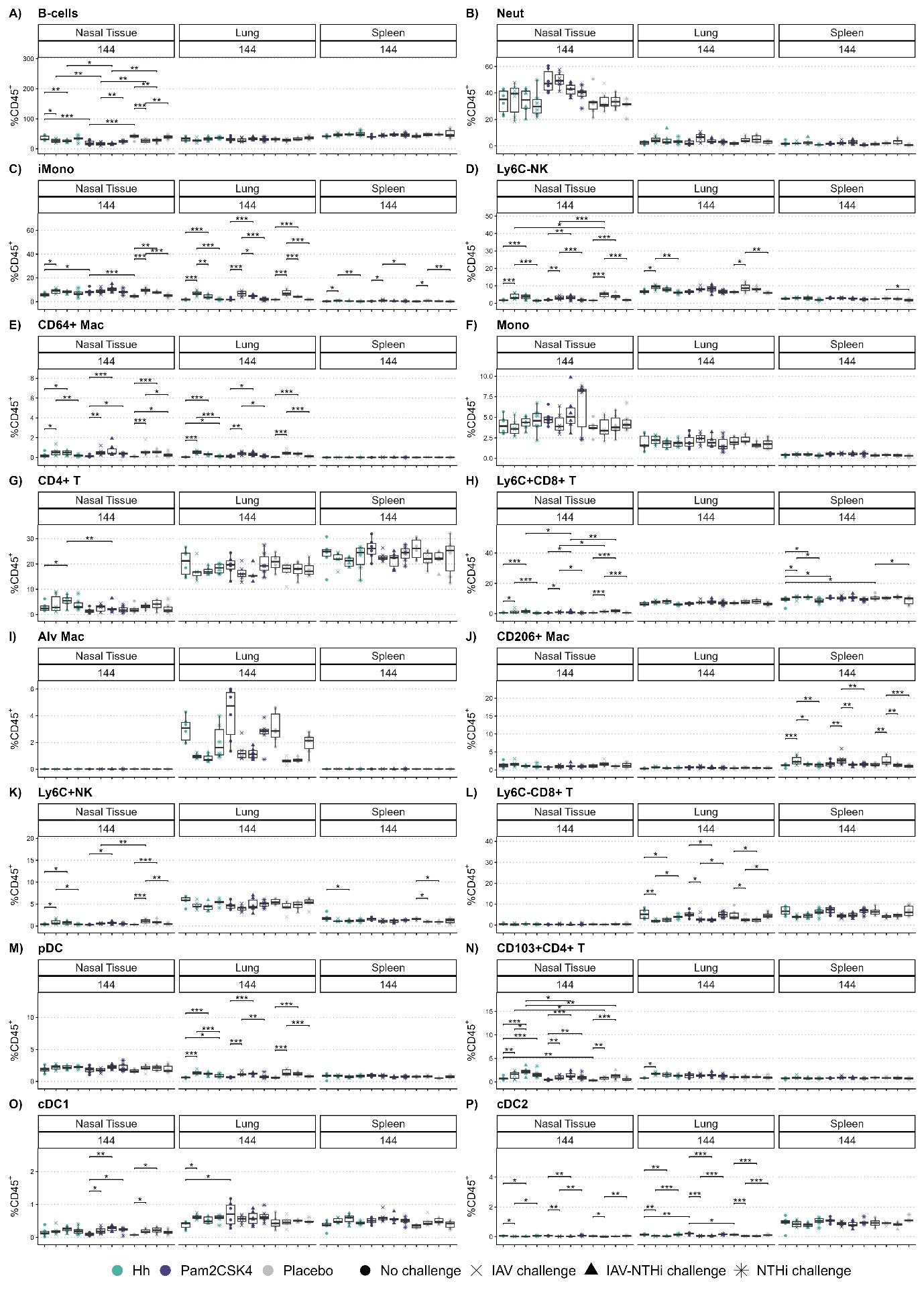


**Supplementary Figure 4**. Boxplots showing the difference between population frequencies of all populations annotated within this dataset across all pre-treatment conditions in the presence or absence of IAV and/or NTHi challenge, tissues, and timepoints. N=6/treatment/tissue/timepoint. (*, ** and *** represent p value <0.05, 0.01 and 0.001, respectively). Neut, Neutrophils; CD64+ Mac, CD64+ M1-like Macrophages; CD206+ Mac, CD206+ M2-like Macrophages; Alv Mac, Alveolar Macrophage; cDC1, Classical Dendritic Cell 1; cDC2, Classical Dendritic Cell 2; pDC, Plasmacytoid Dendritic Cell; CD4+ T, CD4+ T-cells; CD103+CD4+ T, CD103+CD4+ T-cells; Ly6C+CD8+ T, Ly6C+CD8+ T-cells; Ly6C-CD8+ T, Ly6C-CD8+ T-cells; Ly6C+ NK, Ly6C+ NK cells; Ly6C- NK, Ly6C- NK cells; Mono, Classical Monocytes; iMono, Inflammatory Monocytes.

**Supplementary Figure 5**


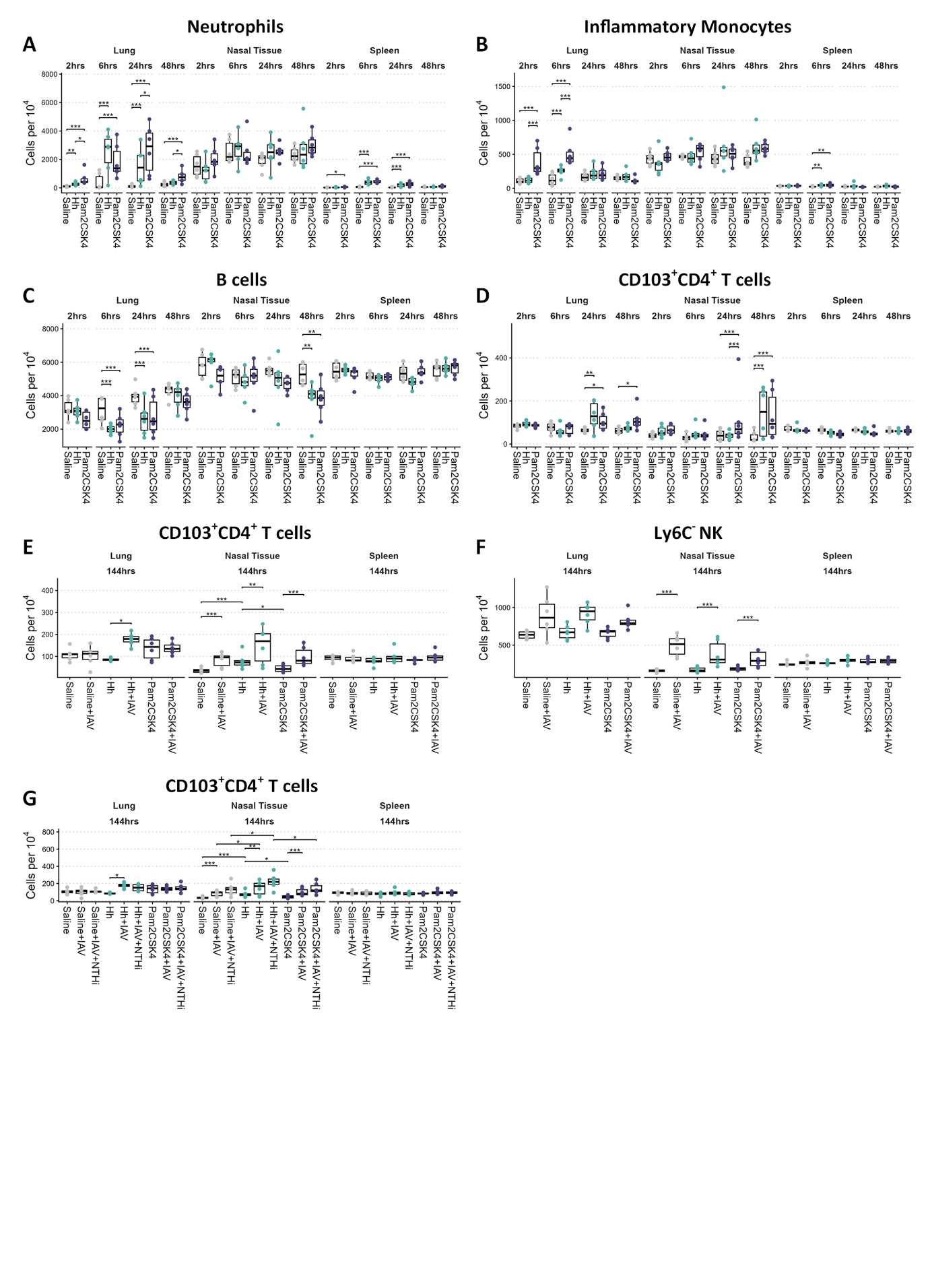


**Supplementary Figure 5**. Boxplots showing the difference between population counts per 10^4^ cells of all populations with frequencies referenced within Figures 3-5. (A-D) Neutrophil, Inflammatory Monocyte, B cell and CD103^+^CD4^+^ T cell counts at 2, 6, 24 and 48 hours after treatment are demonstrated. (E-F) CD103^+^CD4^+^ T cell and Ly6C^-^NK cell counts at 144 hours in the IAV single challenge model are shown. (G) Counts of CD103^+^CD4^+^ T cells at 144 hours in the full IAV+NTHi OM challenge model are shown.

(*, ** and *** represent p value <0.05, 0.01 and 0.001, respectively).

**Supplementary Figure 6.** Gating strategy to identify singlets (FSC-H vs FSC-A), stable acquisition (time gating), autofluorescence-negative populations (AF-1^-^AF-2^-^) and live CD45^+^ events, prior to computational analysis in R.


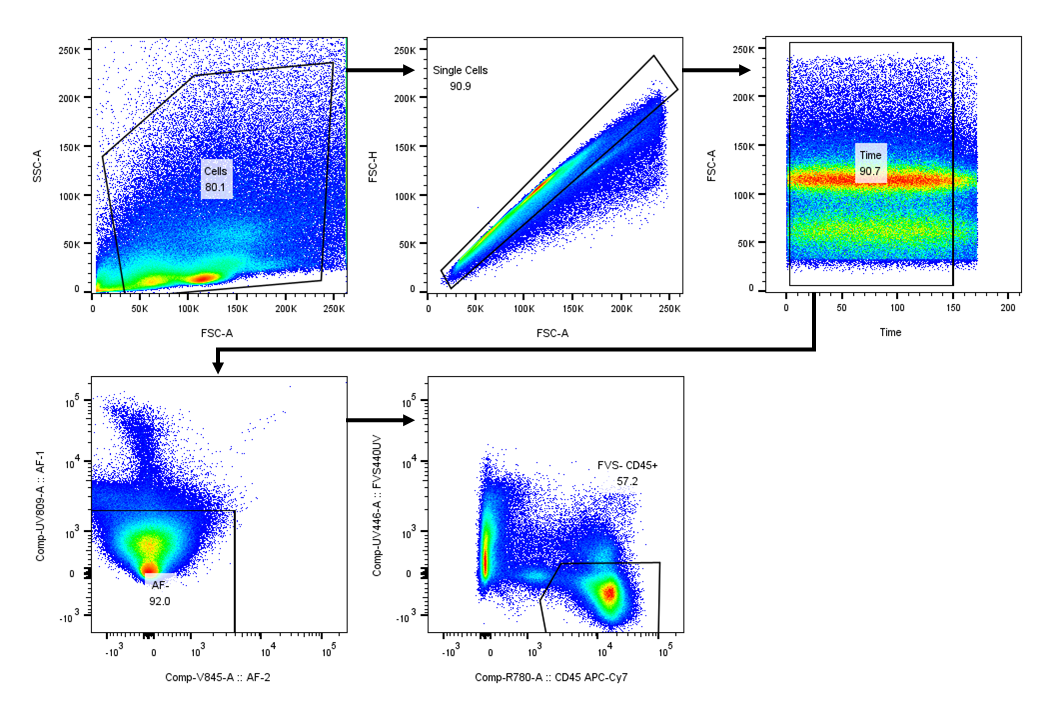


**Supplementary Table 1**. Number of Mice per Treatment per Timepoint.

| ***Treatment*** | ***Timepoint*** | | | | | | | ***Total (M/F)*** |
| --- | --- | --- | --- | --- | --- | --- | --- | --- |
|  | 2h | 6h | 24h | 48h | 72h | 96h | 144h |  |
| Hh | 3M/3F | 3M/3F | 4M/2F | 3M/3F | 3M/3F | 3M/3F | 3M/3F | 22:20 |
| Pam2CSK4 | 3M/3F | 3M/3F | 3M/3F | 3M/3F | 3M/3F | 3M/3F | 3M/3F | 21:21 |
| Saline | 3M/3F | 3M/3F | 3M/3F | 3M/3F | 3M/3F | 3M/3F | 3M/3F | 21:21 |
| Hh+IAV | **-** | **-** | **-** | 3M/3F | 3M/3F | 3M/3F | 3M/3F | 12:12 |
| Pam2CSK4+IAV | **-** | **-** | **-** | 3M/3F | 3M/3F | 3M/3F | 3M/3F | 12:12 |
| Saline+IAV | **-** | **-** | **-** | 3M/3F | 3M/3F | 3M/3F | 3M/3F | 12:12 |
| Hh+NTHi | **-** | **-** | **-** | 3M/3F | 3M/3F | 3M/3F | 3M/3F | 12:12 |
| Pam2CSK4+NTHi | **-** | **-** | **-** | 3M/3F | 3M/3F | 3M/3F | 3M/3F | 12:12 |
| Saline+NTHi | **-** | **-** | **-** | **-** | **-** | **-** | 3M/3F | 3:3 |
| Hh+IAV+NTHi | **-** | **-** | **-** | **-** | **-** | **-** | 3M/3F | 3:3 |
| Pam2CSK4+IAV+NTHi | **-** | **-** | **-** | **-** | **-** | **-** | 3M/3F | 3:3 |
| Saline+IAV+NTHi | **-** | **-** | **-** | **-** | **-** | **-** | 3M/3F | 3:3 |
| ***Total*** | *18* | *18* | *18* | *36* | *36* | *36* | *72* | *234* |

*Influenza A Virus (IAV) administration occurred at 24h; nontypeable *Haemophilus Influenzae* (NTHi) administration occurred at 96h.

**Supplementary Table 2**. Flow cytometry reagents used in this study

| *λ_Ex_* | *Parameter* | *Target* | *Fluorophore* | *Clone* | *Manufacturer* | *Cat No.* | *Lot No.* | *Final Dil.* |
| --- | --- | --- | --- | --- | --- | --- | --- | --- |
| - | - | Brilliant Stain Buffer Plus | - | - | BD Horizon | 566385 | 3199144 | 1:20 |
| 355 | UV379 | Hamster anti-mouse CD11c | BUV395 | HL3 | BD Horizon | 624165 | 335473* | 1:40 |
| 355 | V450 | Live/Dead | FVS440UV | N/A | BD Horizon | 566332 | 4002117 | 1:2000 |
| 405 | V427 | Rat anti-mouse F4/80 | BV421 | BM8 | BioLegend | 123132 | B401780 | 1:80 |
| 405 | V470 | Hamster anti-mouse CD3e | BV480 | 145-2C11 | BD OptiBuild | 624249 | 3335490* | 1:40 |
| 405 | V540 | Rat anti-mouse CD4 | SV538 | GK1.5 | BioLegend | 100486 | B380134 | 1:640 |
| 405 | V595 | Rat anti-mouse Ly6G | BV605 | 1A8 | BD Horizon | 563005 | 3187156 | 1:160 |
| 405 | V660 | Mouse anti-mouse/rat XCR1 | BV650 | ZET | BioLegend | 148220 | B392722 | 1:320 |
| 405 | V710 | Rat anti-mouse CD11b | BV711 | M1/70 | BD Horizon | 563168 | 3227261 | 1:640 |
| 405 | V785 | Rat anti-pDCA-1 (CD317) | BV786 | 927 | BD OptiBuild | 747603 | 3334004 | 1:160 |
| 488 | B510 | Rat anti-mouse Ly6C | FITC | AL-21 | BD Pharmingen | 553104 | 3138565 | 1:80 |
| 488 | B537 | Rat anti-mouse CD8a | RB545 | 53-6.7 | BD Horizon | 569278 | 3308053 | 1:640 |
| 488 | B810 | Rat anti-mouse I-A/I-E | RB780 | 2G9 | BD OptiBuild | 755849 | 3334039 | 1:10,000 |
| 561 | YG585 | Rat anti-mouse CD103 | PE | M290 | BD Pharmingen | 557495 | 3212885 | 1:80 |
| 561 | YG602 | Rat anti-mouse CD45R (B220) | PE-CF594 | RA3-6B2 | BD Horizon | 562290 | 3216017 | 1:160 |
| 561 | YG670 | Rat anti-mouse CD206 | PE-Cy5 | C068C2 | BioLegend | 141740 | B403623 | 1:160 |
| 561 | YG780 | Rat anti-mouse NKp46 (CD335) | PE-Cy7 | 29A1.4 | BioLegend | 137618 | B399934 | 1:40 |
| 637 | R675 | Mouse anti-mouse CD64 | AF647 | X54-5/7.1 | BD Pharmingen | 558539 | 2293025 | 1:320 |
| 637 | R710 | Rat anti-mouse CD19 | APC-R700 | 1D3 | BD Horizon | 565473 | 3180581 | 1:320 |
| 637 | R780 | Rat anti-mouse CD45 | APC-Cy7 | 30-F11 | BD Pharmingen | 557659 | 3264235 | 1:640 |

*BD Horizon™ Custom special-order antibodies

**Supplementary Table 3**. Surface marker classification for each annotated cell type

| ***Cell Phenotype*** | ***Abbreviation*** | ***Marker Classification*** |
| --- | --- | --- |
| Neutrophils | Neut | Ly6G^+^CD11b^+^ |
| CD64^+^ M1-like Macrophages | CD64^+^ Mac | F4-80^+^CD11b^+^CD64^+^I-A/I-E^-^ |
| CD206^+^ M2-like Macrophages | CD206^+^ Mac | F4-80^+^CD11b^+^CD64^+^I-A/I-E^+^CD206^+^ |
| Alveolar Macrophage | Alv Mac | F4/80+CD11c+CD64+ |
| Classical Dendritic Cell 1 | cDC1 | CD11c^+^I-A/I-E^+^XCR1^+^ |
| Classical Dendritic Cell 2 | cDC2 | CD11c^+^I-A/I-E^+^CD11b^+^ |
| Plasmacytoid Dendritic Cell | pDC | B220^+^pDCA-1^+^ |
| CD4^+^ T-cells | CD4^+^ T | CD3e^+^CD4^+^ |
| CD103^+^CD4^+^ T-cells | CD103^+^CD4^+^ T | CD3e^+^CD4^+^CD103^+^ |
| Ly6C^+^CD8^+^ T-cells | Ly6C^+^CD8^+^ T | CD3e^+^CD8^+^Ly6C^+^ |
| Ly6C^-^CD8^+^ T-cells | Ly6C^-^CD8^+^ T | CD3e^+^CD8^+^Ly6C^-^ |
| Ly6C^+^ NK cells | Ly6C^+^ NK | NKp46^+^Ly6C^+^ |
| Ly6C^-^ NK cells | Ly6C^-^ NK | NKp46^+^Ly6C^-^ |
| Classical Monocytes | Mono | Ly6C^+^CD11b^+^CD64^lo^ |
| Inflammatory Monocytes | iMono | Ly6C^+^CD11b^+^CD64^+^I-A/I-E^+^ |
| B-cells | B-cells | B220^+^CD19^+^ |

**Supplementary Table 4.** ARRIVE2.0 checklist

| ARRIVE Essential 10 Checklist | | |
| --- | --- | --- |
| Study design | 1 | For each experiment, provide brief details of study design including:   1. The groups being compared, including control groups. If no control group has been used, the rationale should be stated.   Detailed in Results (lines 293-304); Presented in Figure 1A+B.   1. The experimental unit (e.g. a single animal, litter, or cage of animals).   Detailed in Methods (lines 120-122) |
| Sample size | 2 | 1. Specify the exact number of experimental units allocated to each group, and the total number in each experiment. Also indicate the total number of animals used.   Detailed in Methods (lines 120-122) and Supplementary Table S3   1. Explain how the sample size was decided. Provide details of any *a priori* sample size calculation, if done.   Detailed in Methods (122-123) |
| Inclusion and exclusion criteria | 3 | 1. Describe any criteria used for including or excluding animals (or experimental units) during the experiment, and data points during the analysis. Specify if these criteria were established a priori. If no criteria were set, state this explicitly.   Detailed in Methods (lines 126-127)   1. For each experimental group, report any animals, experimental units, or data points not included in the analysis and explain why. If there were no exclusions, state so.   Detailed in Methods (lines 126-127)   1. For each analysis, report the exact value of n in each experimental group.   Detailed in Supplementary Table S1 and in each figure legend where appropriate. |
| Randomisation | 4 | 1. State whether randomisation was used to allocate experimental units to control and treatment groups. If done, provide the method used to generate the randomisation sequence.   Detailed in Methods (lines 123-124, lines 137-139)   1. Describe the strategy used to minimise potential confounders such as the order of treatments and measurements, or animal/cage location. If confounders were not controlled, state this explicitly.   Detailed in Methods (lines 137-139) |
| Blinding | 5 | Describe who was aware of the group allocation at the different stages of the experiment (during the allocation, the conduct of the experiment, the outcome assessment, and the data analysis).  Detailed in Methods (line 125) |
| Outcome measures | 6 | 1. Clearly define all outcome measures assessed (e.g. cell death, molecular markers, or behavioural changes).   Explicitly detailed throughout Results (lines 322 - 461)   1. For hypothesis-testing studies, specify the primary outcome measure, i.e. the outcome measure that was used to determine the sample size.   N/A – exploratory study |
| Statistical methods | 7 | 1. Provide details of the statistical methods used for each analysis, including software used.   Statistical analyses are detailed in the Methods (lines 253-288).  Computational cytometry analyses are detailed in the Methods (lines 198-222)   1. Describe any methods used to assess whether the data met the assumptions of the statistical approach, and what was done if the assumptions were not met.   N/A |
| Experimental animals | 8 | 1. Provide species-appropriate details of the animals used, including species, strain and substrain, sex, age or developmental stage, and, if relevant, weight.   Detailed in Methods (lines 117-119)   1. Provide further relevant information on the provenance of animals, health/immune status, genetic modification status, genotype, and any previous procedures.   Detailed in Methods (lines 117-120) |
| Experimental procedures | 9 | For each experimental group, including controls, describe the procedures in enough detail to allow others to replicate them, including:   1. What was done, how it was done, and what was used.   Detailed in Methods (lines 130-139)   1. When and how often.   Detailed Results (lines 293-304); Presented in Figure 1A+B.   1. Where (including detail of any acclimatisation periods).   Detailed in Methods (line 120)   1. Why (provide rationale for procedures).   Detailed in the Introduction (lines 93-100) and Results (lines 293-297) |
| Results | 10 | For each experiment conducted, including independent replications, report:   1. Why (provide rationale for procedures).   Detailed in Results at the start of each section, where appropriate (lines 293-297, 353-358, 402-408, 436-437)   1. If applicable, the effect size with a confidence interval.   Fold-changes (relative to saline) are reported throughout the results of the manuscript as a measure of effect size. IQRs are presented as measurement of spread, given limited sample sizes. The raw data detailing individual units are displayed to demonstrate effect size differences and variance; and complete data are included in Supplementary Figures 2-4 |
